# Incentives and Trust Are the Main Drivers of Recruiting Participants in 6 African Countries via Web-Based Environments: A Vignette Survey Experiment

**DOI:** 10.2196/68472

**Published:** 2025-06-25

**Authors:** Henning Silber, Björn Rohr, Jan Priebe

**Affiliations:** 1 Survey Research Center, Institute for Social Research, University of Michigan–Ann Arbor, 426 Thompson Street, Ann Arbor, MI, 48104, United States, 1 734-764-8354; 2 Survey Statistics Team, Department of Survey Design and Methodology, GESIS - Leibniz-Institute for the Social Sciences, Mannheim, Germany; 3 Health Economics Research Group, Bernhard Nocht Institute for Tropical Medicine, Hamburg Center for Health Economics, Hamburg, Germany

**Keywords:** monitoring health outcomes, web-based recruitment, survey methodology, vignette experiment, participation, incentives, study sponsor, topic, sub-Saharan Africa, biomarker

## Abstract

**Background:**

In-person health surveys and biomarker collections (eg, blood testing) provide crucial data to monitor and investigate progress on health outcomes in sub-Saharan Africa. Bearing in mind that administrative sampling frames are often outdated and financial resources can be limited, it is of substantial policy importance to better understand whether recruitment of individuals for in-person health data collection efforts can be accomplished via web-based environments such as social media sites. Yet, there is little methodological research on (1) the feasibility of recruitment through web-based environments and (2) the factors that drive in-person survey participation rates in sub-Saharan Africa countries.

**Objective:**

This study aimed to share our experimental results on the recruitment of individuals from sub-Saharan Africa for participation in in-person, health-related surveys and biomarker collections via Facebook ads and to provide recommendations for future data collections and research.

**Methods:**

We conducted a preregistered 2×4×4 vignette experiment to investigate people’s willingness to participate in in-person health surveys and blood tests. The experiment was part of a web survey (n≈10,600) of individuals recruited via Facebook advertisements that we conducted in early 2023 in 6 sub-Saharan Africa countries (Ghana, Kenya, Nigeria, South Africa, Tanzania, and Uganda). Based on the theories of contextual integrity, economic participation, and social exchange, three factors were varied: (1) the topic (HIV or diabetes), (2) the incentive (US $0, US $2 cash, US $2 voucher, and US $2 lottery), and (3) the sponsor (nongovernmental organization, statistical office, health ministry, or local university).

**Results:**

Overall, we found that a majority of survey participants are willing to participate in in-person health surveys and provide biomarkers (vignette means range between 5.54 and 6.09 on a 1 to 7 scale). First, providing a financial incentive significantly increased the likelihood of being willing to participate (*b*=.180, .188, and .200; all *P*<.001). Second, individuals with high levels of trust in nongovernmental organizations or the health ministry were more likely to be willing to participate (*b*=.086 and .048; both *P*<.001). In contrast, 2 factors (topic and sponsor) showed mainly non-significant effects (*b*=.010, *P*=.63; *b*=.041, *P*=.18; *b*=.042, *P*=.19; *b*=.063, *P*=.05). Other factors that were related to an increase in willingness to participate included fertility levels (having children), risk-taking, having an illness (HIV, diabetes), better general health, social trust, trust in science, survey enjoyment, survey value, and cognitive skills.

**Conclusions:**

Together, the study’s results suggest that using a web-based environment for recruiting health research participants in sub-Saharan Africa can be a viable option and emphasize the importance of adequate compensation and trust in the sponsor. The findings also indicated that several attitudinal but almost none of the sociodemographic variables are systematically related to the willingness to participate in health-related in-person data collection activities.

## Introduction

One of the most significant challenges facing Africa’s health care systems is the scarcity of reliable administrative, routine health data. This lack of data hinders efforts to monitor infectious diseases, allocate medical resources effectively, and implement digital health solutions and evidence-based practices. The root causes of this data scarcity are multifaceted, including inadequate infrastructure, insufficient training capacity, and the heavy workload of health care professionals [1-3].

Consequently, in-person health surveys continue to be an essential tool in sub-Saharan Africa to monitor and investigate health-related attitudes, knowledge, practices, and outcomes. For example, the Demographic and Health Surveys and UNICEF’s (United Nations Children's Fund) Multiple Indicator Cluster Surveys constitute the primary data source to track maternal anemia rates, HIV prevalence rates, child anthropometric outcomes, and skilled birth attendance for the majority of sub-Saharan Africa countries and therefore ultimately inform global progress on these indicators as part of the UN’s Sustainable Development Goals [4,5] and several of the Lancet’s Global Burden of Disease studies [6].

Traditionally, the sampling frame for in-person health surveys in sub-Saharan Africa relies exclusively on population census data. However, due to high mobility rates and infrequent census data collection efforts, these data are often considered to be outdated [7]. Furthermore, given the circumstance that population censuses do not collect health information, the usefulness of these data to particularly target specific groups such as people with disabilities, malaria, or HIV is limited.

Against this backdrop, this study examines whether individuals from the general population in sub-Saharan Africa can be recruited from web-based environments, more specifically the social media platform Facebook, in order to participate in in-person interviews and health data collection (eg, blood tests). Since the recruitment of study subjects for health surveys and data is costly in terms of preparation time and budget, it is important for policy makers and researchers alike to understand (1) whether recruitment of study subjects is feasible and (2) which factors influence individuals’ intention to participate in health-related surveys and data collection; particularly in resource-poor settings such as sub-Saharan Africa. In this context, it should be noted that the willingness to participate in surveys and data collection efforts is on the decline in many countries around the world [8,9]; a worrying trend that makes it even more critical to better understand the factors influencing survey participation.

For Western countries, a sizeable amount of literature already exists that examines recruitment of study subjects from web-based environments. For example, scholars have analyzed the feasibility, determinants, or cost-effectiveness of recruiting individuals using social media and Google ads targeting the general population [10], clinical trial population [11,12], students [13], hard-to-reach populations such as sexually underrepresented groups [14], and populations with specific health profiles such as pregnant women [15] or smokers [16]. Similarly, researchers have explored incentives and other factors that influence individuals’ intentions and decisions to participate in surveys (eg, [17-22]).

To what extent findings from Western countries can be generalized to non-Western contexts is not yet well understood [23]. We believe that the findings from studies conducted in Western countries may not be directly applicable to the African context. Several factors, including limited Internet infrastructure and mobile phone coverage, a significant digital divide, and language barriers, are likely to influence selection effects and participation rates in ways that differ from those in Western countries. Additionally, stigmas surrounding certain diseases, such as HIV/AIDS, and concerns about data security and trust may also vary across regions. Furthermore, the value placed on participating in academic research may differ between Western and many sub-Saharan Africa countries, which could impact response rates. Regarding sub-Saharan Africa and Africa more generally, to our knowledge, no prior study has investigated how survey implementation factors affect the willingness to participate in health surveys. In order to zoom into individuals’ decisions to participate in in-person health surveys and data collection efforts in sub-Saharan Africa, we adopt a sociological perspective and let our empirical analysis be guided by 3 distinct theoretical approaches. First, the theory of contextual integrity [24] suggests that individuals consider some data flows as more and some as less appropriate depending on the specific context or setting. Relevant parameters that constitute a context are the data type, the involved actors, such as the sender and recipient, and the transmission principle. In contrast, the economic participation theory [25-27] suggests that the data or time that an individual provides is a good that can be traded for other goods, such as monetary incentives. Finally, the social exchange theory [28,29] assumes that every interaction is built on social trust. In this perspective, an individual’s participation depends on the level of trust in the researcher or study’s sponsor.

In the present study, we developed and preregistered a vignette experiment, which tests specific assumptions related to these 3 theories. Each vignette provided respondents with information on (1) an in-person general health survey and (2) the collection of biomarkers (blood samples) that were planned to be conducted in their region of residence. Ultimately, we asked respondents whether they would be willing to participate in the survey and blood sampling activities. As part of the vignettes, the information provided to respondents varied along three factors: (1) topic, (2) financial incentive, and (3) sponsor. While the experimental factors topic and financial incentive were both informed by the economic participation theory as psychological participation costs were thought to be higher for more stigmatized diseases (eg, HIV) than for less stigmatized diseases (eg, diabetes), the factor sponsor was informed by the theory of contextual integrity. Data was collected from a web survey recruited via Facebook advertisements in 6 sub-Saharan Africa countries (Ghana, Kenya, Nigeria, South Africa, Tanzania, and Uganda). Respondents were recruited via Facebook advertisements.

## Methods

### Experiment

We conducted a preregistered vignette experiment (or factorial survey experiment; [30]) in which we randomly varied 3 factors based on theoretical considerations. Prior to the data collection process, we estimated for the experiment a minimum sample size of 1280 respondents. Sample size calculations were based on an approximated power analysis using an ANOVA design with repeated measures and within-between interaction, using the software G*Power [31] (input parameters: effect size=0.1, alpha error probability=0.05, power=0.95, number of groups=64, number of measurements=2, nonsphericity correction=1). To meet this minimum sample size requirement for every country, we aimed to achieve 1500 completed interviews per country. The three factors (Table 1) included in the experiment were as follows: (1) topic (HIV, diabetes), (2) financial incentive (none, US $2 cash, US $2 lottery, and US $2 voucher), and (3) sponsor (nongovernmental organization [NGO], statistical office, health ministry, or local university). This resulted in 32 unique vignettes (2×4×4). Individuals were randomized into 1 out of the 32 vignette scenarios. Randomization was done separately for each of the 2 tasks (in-person survey participation vs participation in blood test).

**Table 1.** Factors and levels included in the vignette experiment on recruiting participants via web-based environments for health-related surveys and blood tests. Directly after each vignette, respondents were asked: “How likely would you take part/agree to be tested in the survey/blood test in such a situation?” For the majority, the response category was on a 7-point rating scale, while for a minority of respondents, it related to a 5-point rating scale. Regarding the 7-point scale, response categories were as follows: very likely (7), likely (6), somewhat likely (5), neither likely nor unlikely (4), Somewhat unlikely (3), unlikely (2), and very unlikely (1).

Dimension and level		Wording
Topic	
HIV		Among other health issues the survey will ask questions about HIV. OR The blood test is used to assess the prevalence of HIV in your country.
Diabetes		Among other health issues the survey will ask questions about Diabetes/high level of blood sugar. OR The blood test is used to assess the prevalence of Diabetes/high level of blood sugar in your country.
Sponsor	
NGO^a^		Imagine that the survey is conducted by an NGO.
Statistical office		Imagine that the survey is conducted by the National Statistical Office.
Health ministry		Imagine that the survey is conducted by the Ministry of Health.
Local university		Imagine that the survey is conducted by a local university.
Incentive	
None		You would receive US $0 for your participation in this survey.
US $2 cash		You would receive US $2 for your participation in this survey.
US $2 lottery		You would receive a 1% chance to win US $200 in a lottery for your participation in this survey.
US $2 voucher		You would receive a phone voucher worth US $2 for your participation in this survey.

a NGO: nongovernmental organization.

In the following, we provide an example vignette for the blood test task (parameters: HIV, US $2 cash, statistical office):

*Imagine you participate in a health survey. The survey would take place in person and around the area where you live. At some point during the interview, the interviewer would ask you whether you would be willing to undergo a blood test.* *The blood test is used to assess the prevalence of HIV in your country. Imagine that the survey was being conducted by the National Statistical Office and that the blood test would take place at the location of the interview. You would receive 2 USD for the blood test. The blood test shall follow ethical guidelines. Meaning your data shall be safe, anonymous, and protected from misuse, and the test results should be provided to you. How likely would you be to participate in the blood test in this situation?*

### Data

#### General Overview

The data for this study were from the Africa Health Survey project (AHS). As part of the AHS, we recruited respondents from 6 African countries (Ghana, Kenya, Nigeria, South Africa, Tanzania, and Uganda) via Facebook advertisements. Facebook was banned in Uganda during the time we conducted our web survey. However, we decided to still include Uganda as many citizens in sub-Saharan Africa connect to Facebook with a VPN service, and we wanted to investigate whether conducting a survey with Facebook ads would still be possible under these circumstances. The ads were distributed over 7 weeks from February 7 to March 22, 2023 (Section S1 in Multimedia Appendix 1 contains further implementation details). Individuals who clicked on the Facebook ads were transferred to a web survey (UniPark) that hosted the vignette experiment. For successful completion of the web survey, individuals were offered the chance to win 5GB of mobile data. Further information on the images used and how many respondents took part after seeing an ad containing each of those images by country can be found in Figure S1 and Table S1 in Multimedia Appendix 1.

In total, 10,588 individuals completed our short survey and vignette experiment until the last question included in our analyses. A total of 12,344 additional individuals started the survey but broke off before, so we did include them. Of those 572 came from Ghana, 6077 from Kenya, 1363 from Nigeria, 453 from South Africa, 512 from Tanzania, and 1611 from Uganda. Our analytical sample comprises 21,176 observations (2 observations per respondent; one for participation in a general health survey and one for participation in blood test as part of a health survey). Additional information about the data collection is available in Checklist 1 (CHERRIES checklist).

#### Outcome Variable

The main outcome variable relates to a respondent’s willingness to participate in a given task (survey and blood test). For the vast majority of the sample (n=17,520) the outcome variable was measured on a 7-point rating scale in which “1” refers to "very unlikely" to participate in the survey/blood test and “7” refers to "very likely" to participate. For a minority of the sample, the outcome variable was measured on a 5-point rating scale (n=3656) for which “1” refers to "very unlikely" to participate and “5” to "very likely" to participate. We decided to use different codings of the response variable to allow for testing the validity of results depending on the adopted response scale.

#### Other Measures

The AHS collected various demographic and socioeconomic information on each respondent such as age, gender, marital status, wealth, and parenthood (Tables S2 [variable construction] and S3 [descriptive statistics] in Multimedia Appendix 1 contains further information on our sample). Additional measures related to (1) the stated level of trust regarding the 4 institutions (NGO, statistical office, health ministry, or local university) that were varied within the vignette experiment as well as social and scientific trust, (2) privacy concerns related to the blood test, (3) the respondent’s health status (whether the respondent has been diagnosed with HIV or diabetes), (4) the willingness to take risks, (5) cognitive skills, and (6) survey attitudes.

### Ethical Considerations

This study was approved by the Medical Chamber of the state of Hamburg (#2022‐300276-WF) and the German Institute for Global and Area Studies (#5/2023). All participants who took part in the study consented to the primary and secondary analysis of research data and to providing public access to the dataset. The information was collected using UniPark, stored inside a local, secure server in Germany, and was encrypted at all times. All data collected were anonymized and no identifiable information was collected (names, addresses, contact information, and images). All respondents were offered the chance to enter a draw to win 1 of 12 phone vouchers (approximately US $12 per voucher).

### Analysis

#### Empirical Specification

Our main empirical specification adopts a multivariable linear regression model in which parameters of interest are obtained from ordinary least square (OLS) estimation. Equation 1 below depicts the related regression equation:


(1) $$Y_{ict}=T_{ict}^{\prime }\gamma +X_{ict}^{\prime }\beta +\mu_{c}+\epsilon_{ict}$$

where $Y_{ict}$ refers to the outcome variable for individual i in country c for task t, T is a matrix related to binary variables that capture the 2 tasks (survey vs blood test) and 3 factors (topic, incentive, sponsor), X are potential control variables used in the robustness checks, μ refers to country fixed effects, and ε is the error term. SEs were clustered at the respondent level.

#### Preregistered Hypotheses

The vignette experiment was preregistered at “OSF Registries.” The preregistration laid out explicit hypotheses related to 3 sociological theories of survey participation. Table 2 summarizes these hypotheses that guide the following empirical analysis and discussion.

**Table 2.** Overview of the preregistered hypotheses, related theories, and explanations.

#	Hypothesis	Related theory	Explanations
1	H1: Respondents’ willingness to participate in a survey is higher than their willingness to undergo a blood test.	Economic participation theory	A task with low burden, such as participating in a survey, would lead to a higher participation rate than a task with a higher burden, such as undergoing a blood test. This assumption is in line with the economic participation theory (Biner and Kidd, 1994 [25]; Porter and Whitcomb, 2003 [26]; Leeper, 2019 [27]), which suggests that individuals aim at minimizing their costs, while maximizing their gains.
2	H2: Respondents are more likely to participate in a survey or to undergo a blood test if the disease is less stigmatized.	Economic participation theory	HIV is expected to be more stigmatized than diabetes. Stigmatization involves psychological costs.
3	H3: Promising incentives will increase the willingness for both survey participation and undergoing a blood test.	Economic participation theory	Financial incentives are more likely to trigger economic motives for survey participation.
4	H4: Providing a cash incentive or a lottery cash incentive increases willingness to participate compared with voucher.	Economic participation theory	Direct compensation (compared with vouchers) makes payments more tangible and therefore is more likely to trigger economic motives for survey participation.
5	H5: Local universities as sponsors increase the likelihood of participation compared with governmental institutions or NGOs^a^.	Theory of contextual integrity	Universities are directly related to research and to a lesser extent to (1) other policy fields and (2) corruption. This assumption is in line with theory of contextual integrity (Nissenbaum, 2010 [24]), which suggests that data flaws are likely to be considered more appropriate if they align with perceptions of higher integrity.
6	H6: The higher the level of trust in the respective survey sponsor, the higher the willingness to participate.	Social exchange theory	Trust is considered important to facilitate reciprocal and altruistic behavior and therefore is conducive for social exchange (Dillman, 1978 [28]; Goyder et al, 2006 [29]).
7	H7: The more concerned respondents regarding the privacy of their blood samples, the less likely they are to be willing to undergo the blood test.	Theory of contextual integrity	Privacy concerns influence the sensitivity level of the requested data and define the data sharing context, as they influence several parameters, which are directly related to the appropriateness of a data flow.
8	H8: If a respondent is HIV positive or was diagnosed with diabetes, the person is less likely to participate.	Theory of contextual integrity	Being diagnosed with the respective disease makes a research topic more sensitive and affects the context of the data sharing request.

a NGO: nongovernmental organization.

## Results

### Main Results

Table 3 shows descriptive results for each vignette. Overall, we observe a high willingness to participate in both the survey and the blood test. The average willingness to participate ranged from 5.54 (survey on HIV, conducted by the National Statistical Office, no incentive offered) to 6.09 (survey on HIV, conducted by the Ministry of Health, incentive to have a 1% chance to win US $200 in a lottery). In total, 6 vignettes showed values of 6.00 or higher in terms of the willingness to participate in either survey or blood test. Each of those 6 vignettes included financial incentives (either as a lottery or cash incentive).

**Table 3.** Mean levels of reported willingness to participate in surveys and blood tests by vignette.

#	Task	Topic	Organization	Incentive	Mean (SD)	Median	95% CI		Observations
1	Blood Test	Diabetes	Local University	US $2	5.74 (1.74)	6	5.55-5.92		359
2	Blood Test	Diabetes	Local University	US $2 voucher	5.81 (1.58)	6	5.64-5.98		341
3	Blood Test	Diabetes	Local University	Chance	5.87 (1.58)	6	5.71-6.03		371
4	Blood Test	Diabetes	Local University	No incentive	5.78 (1.55)	6	5.60-5.95		317
5	Blood Test	Diabetes	Ministry of Health	US $2	6.04 (1.37)	6	5.89-6.19		319
6	Blood Test	Diabetes	Ministry of Health	US $2 voucher	5.85 (1.62)	6	5.67-6.02		336
7	Blood Test	Diabetes	Ministry of Health	Chance	5.94 (1.46)	6	5.78-6.10		325
8	Blood Test	Diabetes	Ministry of Health	No incentive	5.80 (1.71)	7	5.62-5.99		336
9	Blood Test	Diabetes	NGO^a^	US $2	5.76 (1.61)	6	5.57-5.96		268
10	Blood Test	Diabetes	NGO	US $2 voucher	5.95 (1.49)	6	5.80-6.10		367
11	Blood Test	Diabetes	NGO	Chance	5.79 (1.61)	6	5.62-5.97		321
12	Blood Test	Diabetes	NGO	No incentive	5.76 (1.73)	6	5.58-5.94		356
13	Blood Test	Diabetes	Statistical Office	US $2	5.83 (1.57)	6	5.65-6.01		283
14	Blood Test	Diabetes	Statistical Office	US $2 voucher	5.77 (1.6)	6	5.60-5.94		335
15	Blood Test	Diabetes	Statistical Office	Chance	5.72 (1.63)	6	5.53-5.90		313
16	Blood Test	Diabetes	Statistical Office	No incentive	5.76 (1.54)	6	5.60-5.92		354
17	Blood Test	HIV	Local University	US $2	5.70 (1.8)	6	5.50-5.90		311
18	Blood Test	HIV	Local University	US $2 voucher	5.84 (1.6)	6	5.67-6.02		329
19	Blood Test	HIV	Local University	Chance	5.70 (1.79)	6	5.51-5.90		330
20	Blood Test	HIV	Local University	No incentive	5.67 (1.7)	6	5.49-5.86		316
21	Blood Test	HIV	Ministry of Health	US $2	5.78 (1.63)	6	5.61-5.96		333
22	Blood Test	HIV	Ministry of Health	US $2 voucher	5.81 (1.63)	6	5.63-5.99		320
23	Blood Test	HIV	Ministry of Health	Chance	6.00 (1.46)	7	5.84-6.16		327
24	Blood Test	HIV	Ministry of Health	No incentive	5.74 (1.67)	6	5.57-5.91		365
25	Blood Test	HIV	NGO	US $2	5.93 (1.51)	6	5.77-6.10		330
26	Blood Test	HIV	NGO	US $2 voucher	5.90 (1.53)	6	5.73-6.07		320
27	Blood Test	HIV	NGO	Chance	5.85 (1.57)	6	5.68-6.01		337
28	Blood Test	HIV	NGO	No incentive	5.72 (1.7)	6	5.54-5.91		327
29	Blood Test	HIV	Statistical Office	US $2	5.90 (1.51)	6	5.75-6.05		378
30	Blood Test	HIV	Statistical Office	US $2 voucher	5.92 (1.58)	7	5.75-6.09		334
31	Blood Test	HIV	Statistical Office	Chance	5.93 (1.53)	6	5.76-6.09		335
32	Blood Test	HIV	Statistical Office	No incentive	5.84 (1.49)	6	5.67-6.01		295
33	Survey	Diabetes	Local University	US $2	5.90 (1.56)	6	5.72-6.07		307
34	Survey	Diabetes	Local University	US $2 voucher	5.95 (1.52)	6	5.78-6.11		335
35	Survey	Diabetes	Local University	Chance	5.91 (1.58)	7	5.74-6.08		337
36	Survey	Diabetes	Local University	No incentive	5.67 (1.68)	6	5.49-5.85		335
37	Survey	Diabetes	Ministry of Health	US $2	5.82 (1.71)	7	5.62-6.01		303
38	Survey	Diabetes	Ministry of Health	US $2 voucher	5.96 (1.56)	7	5.79-6.13		320
39	Survey	Diabetes	Ministry of Health	Chance	5.81 (1.58)	6	5.65-5.98		349
40	Survey	Diabetes	Ministry of Health	No incentive	5.60 (1.69)	6	5.43-5.78		359
41	Survey	Diabetes	NGO	US $2	5.96 (1.48)	6	5.81-6.12		337
42	Survey	Diabetes	NGO	US $2 voucher	5.99 (1.46)	7	5.84-6.15		350
43	Survey	Diabetes	NGO	Chance	5.88 (1.53)	6	5.72-6.04		339
44	Survey	Diabetes	NGO	No incentive	5.58 (1.74)	6	5.39-5.77		316
45	Survey	Diabetes	Statistical Office	US $2	5.86 (1.52)	6	5.7-6.03		317
46	Survey	Diabetes	Statistical Office	US $2 voucher	5.85 (1.54)	6	5.68-6.01		336
47	Survey	Diabetes	Statistical Office	Chance	6.03 (1.49)	7	5.86-6.20		301
48	Survey	Diabetes	Statistical Office	No incentive	5.67 (1.67)	6	5.50-5.85		353
49	Survey	HIV	Local University	US $2	5.88 (1.59)	6	5.71-6.05		334
50	Survey	HIV	Local University	US $2 voucher	5.87 (1.6)	6	5.69-6.04		319
51	Survey	HIV	Local University	Chance	5.88 (1.57)	6	5.71-6.04		337
52	Survey	HIV	Local University	No incentive	5.65 (1.64)	6	5.48-5.83		356
53	Survey	HIV	Ministry of Health	US $2	6.03 (1.42)	7	5.88-6.19		332
54	Survey	HIV	Ministry of Health	US $2 voucher	5.87 (1.61)	7	5.69-6.04		330
55	Survey	HIV	Ministry of Health	Chance	6.09 (1.39)	7	5.94-6.25		320
56	Survey	HIV	Ministry of Health	No incentive	5.66 (1.69)	6	5.48-5.85		333
57	Survey	HIV	NGO	US $2	5.96 (1.43)	6	5.80-6.12		313
58	Survey	HIV	NGO	US $2 voucher	5.99 (1.46)	7	5.83-6.16		306
59	Survey	HIV	NGO	Chance	5.79 (1.67)	6	5.61-5.97		326
60	Survey	HIV	NGO	No incentive	5.63 (1.8)	6	5.44-5.82		337
61	Survey	HIV	Statistical Office	US $2	6.01 (1.46)	7	5.86-6.17		347
62	Survey	HIV	Statistical Office	US $2 voucher	5.98 (1.49)	7	5.82-6.14		330
63	Survey	HIV	Statistical Office	Chance	5.84 (1.73)	7	5.65-6.02		341
64	Survey	HIV	Statistical Office	No incentive	5.54 (1.7)	6	5.36-5.73		333

a NGO: nongovernmental organization.

**Figure 1.**
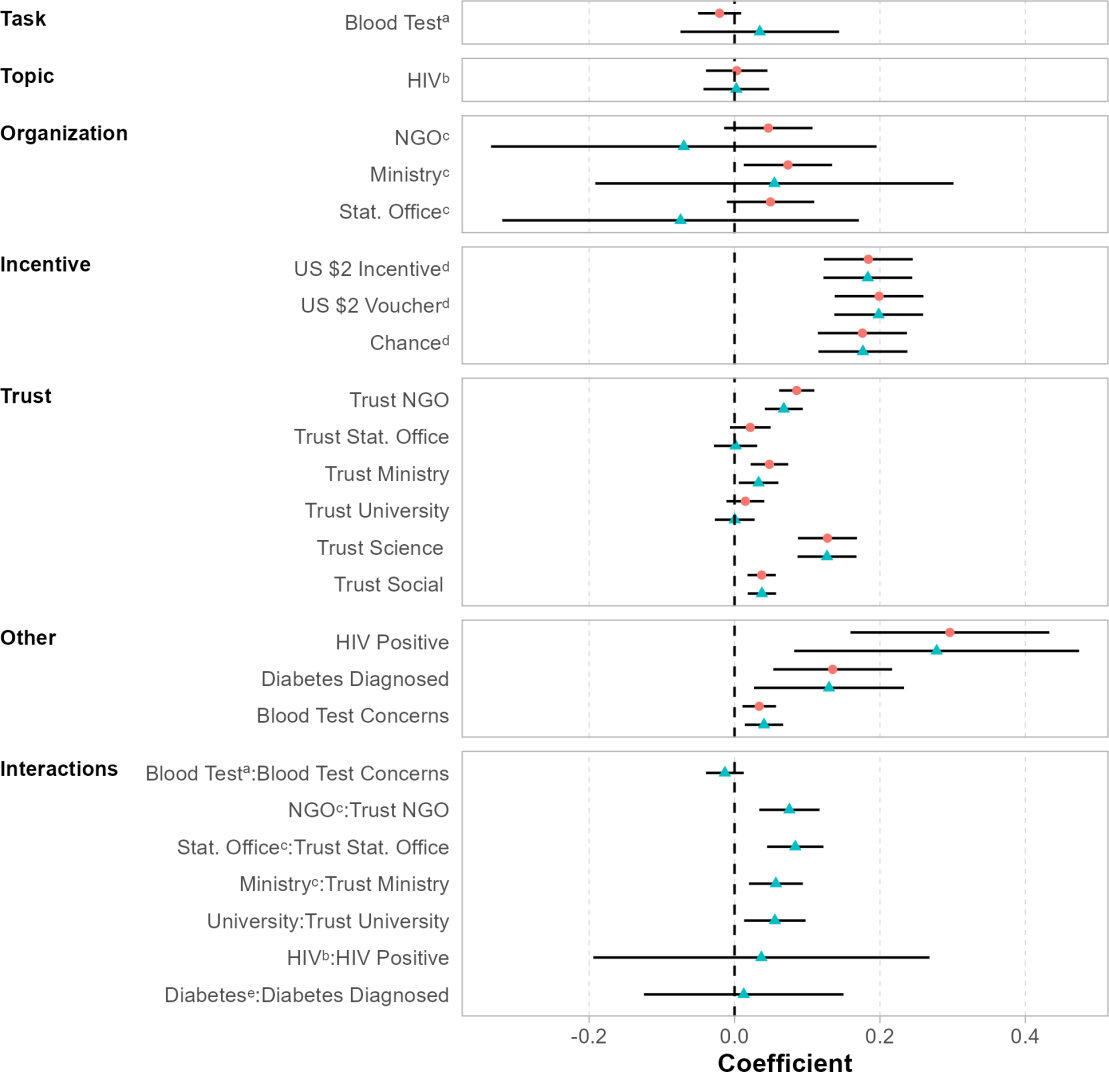
Determinants of survey and blood test participation: Main effects of the vignette experiment and hypotheses. Results are obtained from ordinary least square estimations. All regression specifications included country-fixed effects. Model 1 (in red) includes the displayed variables without any interaction effects, while model 2 (in blue) includes interaction effects for trust and health status (HIV, diabetes) variables. Left-out reference categories are as follows: ªSurvey, ᵇDiabetes, ᶜUniversity, ᵈNo Incentive, ᵉHIV. SEs are clustered at the respondent level. The displayed CIs are at the 95% significance level. Table S4 in Multimedia Appendix 1 contains exact parameter values.

Turning toward the multivariate OLS analysis, Figure 1 depicts results that relate to the 2 tasks (survey vs blood test) and all 3 factors. In the following, we discuss results from model specifications without any interaction effects (Model 1) that are related to hypotheses 1 to 5 from the preanalysis plan (Table S4 [contains the underlying regression] in Multimedia Appendix 1). In contrast to our initial expectations, we do not find any evidence that individuals’ willingness differs across task types (Hypothesis #1; survey vs blood test), the topic of the task (Hypothesis #2; diabetes vs HIV), and the sponsor (Hypothesis #5; local university vs Ministry of health vs statistical office vs NGO). A minor exception concerns vignettes related to the Ministry of Health as a sponsor of the survey or blood test. Having the Ministry of Health as a sponsor increases the probability of participating in surveys or blood tests by about 6.3 percentage points versus the reference category (local universities). However, having the Ministry of Health as a sponsor is not advantageous over the 2 other categories (NGOs, National Statistical Office). Further, we find strong evidence in support of the view that financial incentives matter for participation in surveys and blood donation tasks. On average, including financial incentives increases the willingness to participate in surveys or blood tests by about 22 to 26 percentage points. In particular, the findings suggest that participants would be more willing to participate in health-related in-person data collection activities if they are financially rewarded which is in line with Hypothesis #3 and the economic participation theory. Yet, we do not observe statistically significant differences between the 3 financial incentive groups (no support of Hypothesis #4; cash vs lottery vs voucher incentive).

### Robustness Checks

We conducted a number of robustness checks to test for the sensitivity of our main results (Tables S5 to S10 in Multimedia Appendix 1). By and large, we find that our results do not change once (1) using ordered logit regressions compared with OLS (Table S5 in Multimedia Appendix 1), (2) re-estimating our main specification using alternative covariate specifications (Table S6 in Multimedia Appendix 1), (3) using a lasso regression and double machine learning specification (Table S7 in Multimedia Appendix 1), (4) adopting a multilevel random effects model (Table S8 in Multimedia Appendix 1), (5) dropping observations in which individuals fail any of the two included survey attention checks (Table S9 in Multimedia Appendix 1), and (6) dropping observations for which the outcome variable was based on the simplified 5-point rating scale (Table S10 in Multimedia Appendix 1). Additionally, we conducted a further analysis to assess the robustness of our results, in which we found that the results were similar considering individual countries when comparing them to the pooled results presented in the main text (Figures S2 to S4 in Multimedia Appendix 1).

### Extension

Next, we examine interaction effects (Model 2 in Figure 1) that relate to hypotheses #6 to #8. In contrast to Hypothesis #7, we find that individuals with elevated privacy concerns seem to be more willing to participate in health-related data collection efforts (*b*=.034). Moreover, and related to Hypothesis #8, we observe that opposite to our hypothesis, individuals with their own health concerns (being HIV positive, being diabetic) are substantially more likely to state their willingness to participate in health-related data collection efforts (*b*=.296 and *b*=.135). Though not preregistered, we believe that this finding can be reconciled with both the economic participation and the social exchange theory. First, individuals who are diagnosed with severe medical conditions (HIV or diabetes) are likely to routinely follow medical check-ups and perceive participation in health surveys and health data collection activities as beneficial for themselves (and others). Second, if they are regularly in need of medical care by nurses or medical doctors, they might be inclined to positively reciprocate by participating in health research.

**Figure 2.**
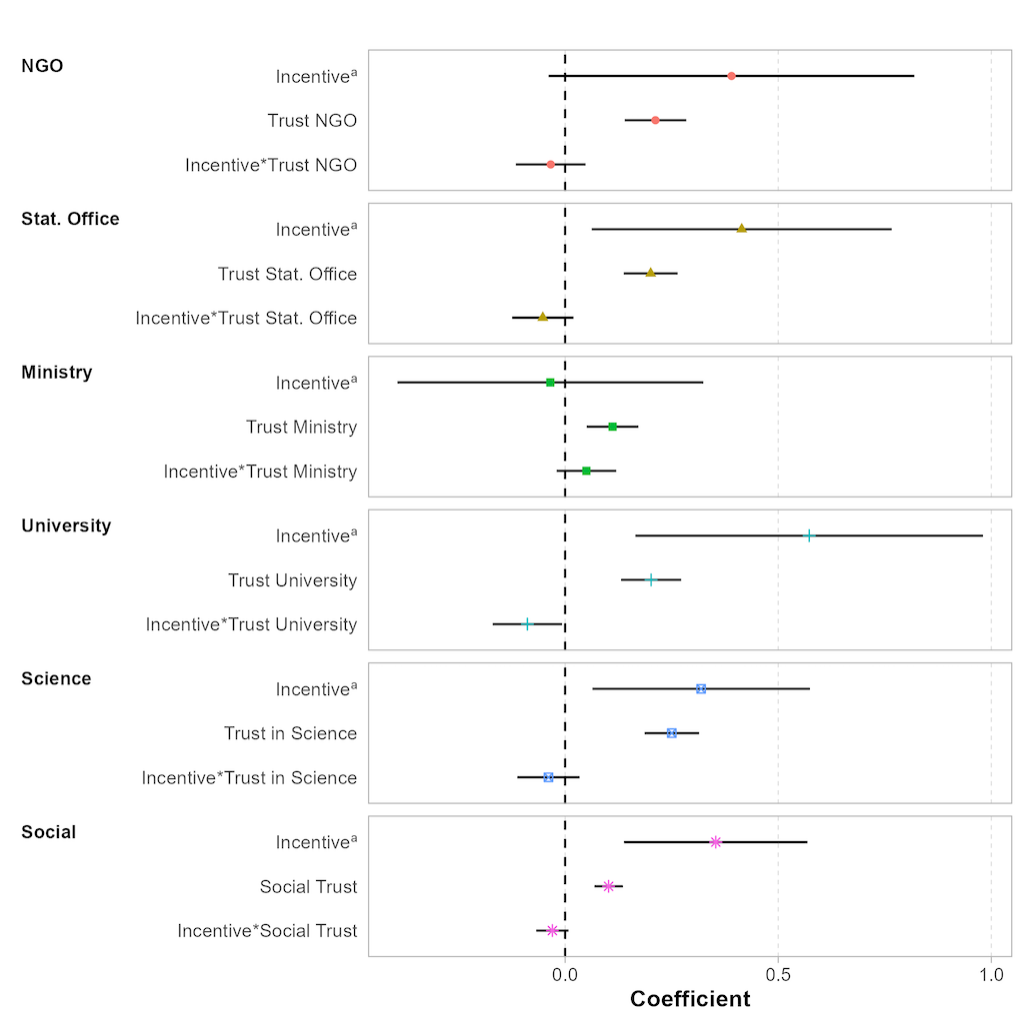
Determinants of survey and blood test participation: Interaction between trust in institutions and incentives. Results are obtained from ordinary least square estimations. Estimations were run separately for each of the 6 trust measures. ^a^The left-out category in each model is the “no incentive” category. All regression specifications included country-fixed effects and the following covariates: binary variables on the survey topic and task. SEs are clustered at the respondent level. The displayed CIs are at the 95% significance level. Table S11 Multimedia Appendix 1 contains exact parameter values.

Last, and in line with Hypothesis #6, trust relationships appear to play an important role, albeit in a very context-dependent manner. For 4 trust indicators (NGOs, Ministry of Health, trust in science, social trust), we observe a clear positive relationship between higher levels of trust and the willingness to participate in surveys or blood tests (Model 2 in Figure 1). In contrast, for 2 trust indicators (National Statistical Office, local universities), we find that these trust indicators are not related to a higher willingness to participate in surveys or blood tests. To better understand the role of trust in our context, we present in Figure 2 results from additional regression specifications that include an interaction effect between trust and incentives. These interaction effects help explore whether these factors are substitutes or complements (eg, re-enforcing each other). Once controlling for the interaction between incentives and trust, we find that higher levels of trust are positively related to the willingness to participate in surveys across all 6 trust measures. In line with this finding, we observe for the 2 institutions for which the trust variable was statistically nonsignificant without adding the interaction (Figure 1), a crowding-out pattern between financial incentives and trust. The related coefficient on the interaction effect is always negative and statistically significant at the 5 percent (local universities) or 10 percent level (National Statistical Office). In general, however, we do not find strong evidence for a crowding-out or crowding-in among trust and incentives. Specifically, the figure suggests that incentives mostly cannot replace trust in the sponsor and vice versa.

**Figure 3.**
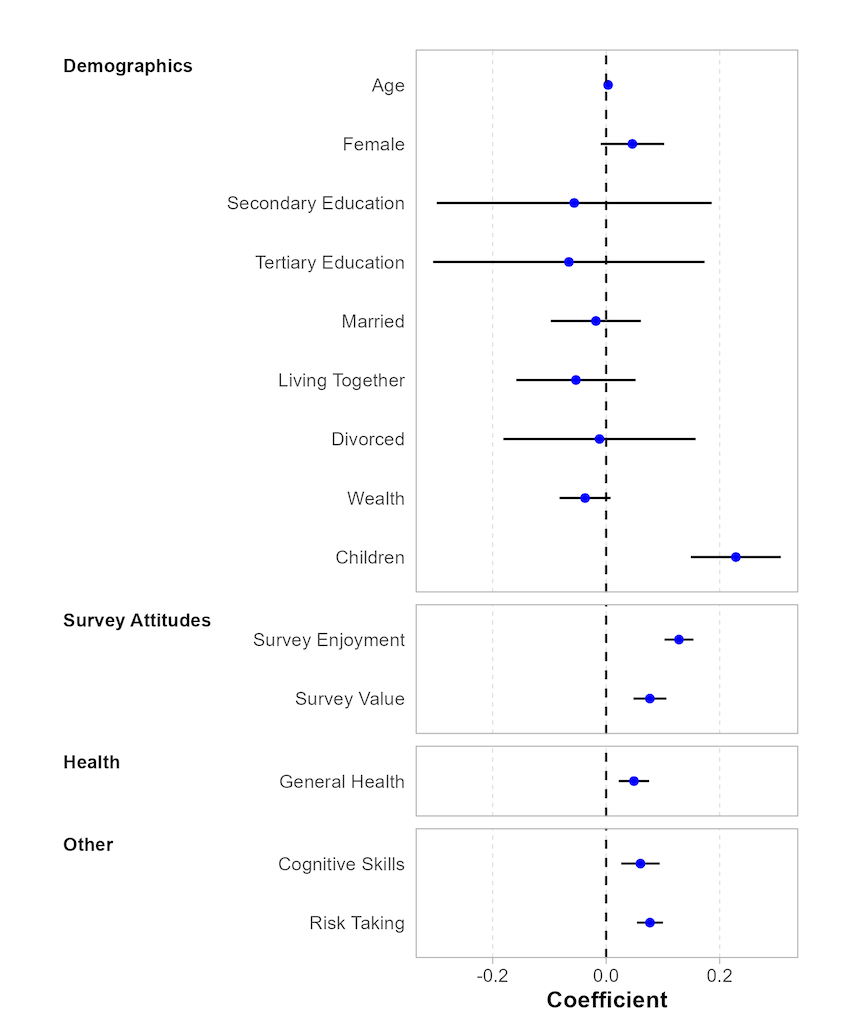
Determinants of survey and blood test participation: Role of background variables. Results are obtained from ordinary least square estimations. All regression specifications included country-fixed effects and the following covariates: binary variables on each vignette parameter (topic, sponsor, and incentive) and task (survey vs blood test). SEs are clustered at the respondent level. The displayed CIs are at the 95% significance level. Table S12 in Multimedia Appendix 1 contains exact parameter values.

Our analysis concluded by examining determinants of the willingness to participate in health surveys and blood tests that are not directly related to factors varied in the vignette. Specifically, we explore in Figure 3 (Table S12 in the Multimedia Appendix 1) the role of demographic and socioeconomic factors and other background variables. Regarding demographics, we found a positive and statistically significant effect of having children on the willingness to participate in health-related, in-person data collection efforts (*b*=.229), whereas we did not find significant effects related to age, education, gender, marital status, and wealth of the study subject. We speculate that the positive and statistically significant coefficient of having children might be reflective of economic and social exchange motives. Bearing in mind that our sample population is relatively young and healthy, many individuals might have their first important contact with nurses and medical doctors when they become parents, which, ceteris paribus, might positively affect individuals’ perceptions of their own benefit from health care and reliable information for health-policy decision-making (economic exchange theory). Likewise, exposure to nurses and doctors at the time of birth might positively influence trust in these actors and lead to a higher willingness to participate in health research (social exchange theory). With respect to the other background variables, we show that the willingness to take risks, generally better health, possessing higher cognitive skills, and positive overall survey enjoyment and value increased the willingness to participate.

## Discussion

### Principal Findings

This article presents results from a preregistered vignette experiment on health research participation within a web survey implemented in 6 African countries. The design was developed drawing from the theory of contextual integrity [24], the economic participation theory [25-27], and the social exchange theory [28,29]. Regarding the 8 hypotheses, Hypothesis 3, which suggested a higher willingness when an incentive was promised, and Hypothesis 6, which suggested a positive effect of trust in the respective sponsor, were supported. In contrast, we did not find the expected effects regarding H1 (task), H2 (topic), H4 (difference across incentives), H5 (difference across sponsors), H7 (privacy concerns), and H8 (HIV or diabetes diagnosis). Notably, for H7 and H8, we found effects opposite to the expected directions, as respondents who had more privacy concerns or were diagnosed with the respective diseases were more likely to participate, possibly due to their personal involvement due to the diagnosis and because privacy concerns might be less relevant for participation than other reasons [32].

Because of the positive effects of the incentives and levels of trust regarding study participation, we tested whether those effects are independent or whether they multiply with one another. Results showed no interaction but independent effects, suggesting that the study’s extrinsic (incentives) and intrinsic motives (trust levels) individually impact survey participation. Notably, not only trust in the respective research organization but also the levels of social trust and trust in science affected willingness to participate.

Of the other variables, having children, cognitive skills, risk-taking, general health, and positive survey attitudes affected the willingness to participate, whereas sociodemographic variables did not show a statistically significant effect on participation levels. Those results suggest that mainly attitudinal and topic-related (ie, health) impact the participation likelihood, while demographic variables seem to be less relevant. Similar to the trust variables, respondents with positive survey attitudes and positive associations with respect to the survey topic are more likely to participate. Specifically, the positive effect of general health is notable, as we found also that respondents diagnosed with HIV or diabetes were more likely to participate. While this seems perhaps to be contradictory, both effects may suggest that an individual, who perceives a survey as more relevant, is more likely to participate, either because the survey is exactly about the disease the person has or because someone with good health is more interested in answering questions about this topic. In contrast, not being diagnosed with HIV or diabetes does not mean that the person is necessarily in good general health.

### Comparison With Prior Work

When comparing our main results to previous work from Western countries, our study confirms that monetary incentives are a strong driver for participation in surveys [33-35] and other data collections [21,36,37]. In contrast to most previous work [33,34,38], we found that a lottery incentive increased the participation likelihood. With respect to topic interest, our study adds to prior experimental evidence about topic interest [39,40] and the survey sponsor [41], showing mixed results regarding their direct effects.

When comparing our findings with respect to interaction effects reported in prior research, we replicated the effects of trust in the survey sponsor [22,42], but did not find the previously identified negative effect of higher privacy concern on participation rates [22,43-45]. With respect to participants’ own health concerns, our finding of higher participation willingness is in line with a recent experimental study which showed that individuals diagnosed with more medical conditions were more likely to share their health data [46]. This finding could mean that individuals with higher health concerns care more about the topic and are therefore more likely to engage in related research. While our survey did not show previously identified demographic effects, such as higher participation of women [47-49] and individuals with higher levels of education [37,50,51], we replicated higher participation rates of individuals with more positive attitudes toward surveys [21,52].

### Implications

From a theoretical perspective, the study’s results underline the relevance of all 3 theories. The economic participation theory was supported, as incentives increased the likelihood of participation. The social exchange theory was supported because trust levels were significantly related to the willingness to participate. While our hypotheses were not supported, the theory of contextual integrity was relevant as both privacy concerns and being diagnosed with HIV or diabetes affected respondents’ willingness to participate. Those results align with the perspective that survey participation is a complex social activity of which the economic aspect is a part, but it goes beyond that by constituting a social situation in which trust and other context-specific parameters affect whether an individual is willing to participate.

From a practical point of view, the study showed that several results from other countries are relevant to the African context. As previous research suggested, all 3 theories and their related constructs explain participation willingness. However, there also seem to be specifics regarding the African context. We did not find the expected variations across different incentives and between different sponsors. While this result could have been due to the hypothetical situation, it could also hint at the specifics of the region, in which requests to participate in research are less common than in Western countries so that other incentives (eg, lottery and voucher) are similarly successful as direct cash incentives. More generally, the study shows that individuals from various demographic groups are willing to participate via web-based environments, making this way of collecting data a real alternative to more traditional methods. However, the effects of trust levels, attitudes, and topical variables illustrate, at the same time, that there are certain groups of respondents that are less likely to participate. Including a sufficiently large number of respondents from those groups will be a crucial task for study designers who would like to use web-based environments for health research.

While it is not our primary research question, the study design and particularly the test of H8 allows us to draw initial conclusions on the intent to participate of respondents diagnosed with HIV or diabetes compared with respondents who are not diagnosed with those diseases. For both outcome variables, the survey participation and the blood test, respondents who were diagnosed with at least one of the diseases reported a higher participation willingness than respondents who were not positively diagnosed (Model 1 in Figure 1 and Figure S4 in Multimedia Appendix 1). These results are promising for research that specifically focuses on those health issues since the relevant groups did not indicate high hesitancy to take part, which will be crucial for generalizable conclusions on those vulnerable groups. Further research with a specific focus on those groups is needed to develop an optimal recruitment design to conduct health research among individuals diagnosed with the respective diseases via web-based platforms and to compare those results to alternative data collection strategies and other available data sources.

### Deviation From the Preregistration

We deviated from the preregistration plan regarding the number of participants per country (ie, 1500). This was done due to an unexpectedly high variation in the number of participants per US $1 spent. During the first 3 weeks of data collection, Kenya outperformed all other countries, while Nigeria and Uganda also reached relatively high participation numbers. In contrast, the data collection was less successful in Ghana, South Africa, and Tanzania in which we would not have reached the 1500 respondents target even if we had continued as originally planned. Therefore, after 3 weeks, we focused the remaining budget on countries that were more cost-effective, regarding the number of participants. While we do not know exactly why Kenya performed so well, this seems to be a common finding, as other research conducted via Facebook advertisement in sub-Saharan Africa also focuses on this country [53-55].

A second deviation from the preregistration plan concerns our experimental design and related analyses. To simplify the experimental design, we decided against randomization by task after the preregistration, leading to a 2×4×4 design instead of a 2×2×4×4 design of the vignette experiment. In line with that adjustment and because the results of separate models for each of the 2 tasks are similar to each other and the pooled analyses (Figure 1 and Figure S5 in Multimedia Appendix 1), we decided to report the pooled results in the manuscript. However, a notable difference between the results of the 2 tasks is that the effect sizes for financial incentives on participation in blood tests are about half as large (and occasionally lose significance) compared with the effects on participation in a survey, which are always highly significant (Figure S5 in Multimedia Appendix 1).

### Limitations

Our study has several limitations and opportunities for further research. First, we only included 6 African countries, and future research has to show whether the results apply to other countries as well. Second, recruitment strategies via social media sites have their caveats. For example, related research [56] shows that the respondents of our vignette experiment are comparable to the general population with respect to a number of sociodemographics such as respondent’s gender, age, and region of living. However, this research also shows that respondents are more likely to be single (not married), have higher daily internet use, and possess higher levels of education compared with the general population. Those results should especially be taken into consideration regarding the generalizability of our findings with respect to the association between the willingness to participate and sociodemographic variables (see Figure 3). It was also found that bivariate and multivariate estimates, which we focus on in this study, were overall less biased compared with the general population than univariate estimates [56]. To what extent a more representative pool of respondents can be generated by optimizing the ads, especially the images that are shown to social media users, remains to be seen and appears to be a fruitful avenue for future research. Third, and regarding the experiment, we have to note that we only asked for hypothetical willingness to participate. While a large body of prior research (eg, [57-59]) has documented that intended behavior and actual behavior are strongly connected, it would be worthwhile to repeat the study using a setting in which individuals are recruited via the web for an actual health survey that involves the real-world collection of biomarkers. Fourth, our research presents only a first step toward a better understanding of how web-based recruitment for in-person health surveys can be optimally designed in sub-Saharan Africa. We believe that important follow-up research could, among others, further explore whether specific target groups (eg, people with HIV infections) can be effectively recruited via social media outreach activities. Previous research has shown that Facebook surveys might be feasible to recruit special populations in Western countries, for example, HIV-positive individuals [60], kidney transplant recipients [61], or cannabis users [62,63], but further research is needed particularly for special populations in other regions like sub-Saharan Africa.

### Conclusion

The results suggest that using web-based environments to recruit respondents for health-related data collections, such as for surveys and blood tests, can be a viable option in the sub-Saharan Africa region. Furthermore, our results provide first insights into important design parameters that seem to be critical in affecting the success of web-based recruitment strategies via Facebook ads and could serve as an initial blueprint. First, financial incentives matter, as promising rewards were related to a substantially higher willingness to participate in in-person health data collection activities. Second, trust in the sponsor behind the in-person health data efforts plays an important role with higher levels of trust leading to (1) a higher absolute number of persons who are willing to participate, and (2) selection effects with persons who distrust a specific sponsor being less likely to be willing to participate. Since trust levels differ substantially for different types of institutions and sponsors, the choice of sponsor should be considered carefully for the respective context. Beyond that, the study’s findings also indicated that several attitudinal but almost none of the sociodemographic variables are systematically related to the willingness to participate in health-related in-person data collection activities. Given that the Demographic and Health Surveys program, an important data source in the sub-Saharan Africa region, was recently discontinued, new data collection methods and recruitment strategies are likely to become more important in the near future.

## Supplementary material

10.2196/68472 Multimedia Appendix 1 Survey implementation, and supplementary tables and figures.

10.2196/68472 Checklist 1 CHERRIES checklist.

## Abbreviations

AHS: Africa Health Survey
NGO: nongovernmental organization
OLS: ordinary least square
UNICEF: United Nations Children's Fund
