# Supplementary material for: Incentives and Trust Are the Main Drivers of Recruiting Participants in 6 African Countries via Web-Based Environments: A Vignette Survey Experiment
Source: J Med Internet Res. 2025 Jun 25;27:e68472. doi: 10.2196/68472 (PMC12220199; doi:10.2196/68472)
Supplement: Multimedia Appendix 1 [file jmir-v27-e68472-s001.pdf]

Online Appendix for the manuscript "Incentives and Trust Are the Main Drivers of Recruiting Participants in 6 African Countries via Web-Based Environments: A Vignette Survey Experiment"

S1: Additional explanations on survey implementation

Recruitment of survey respondents

For the recruitment, 42 different advertisement sets (adsets) were used within Meta's ads manager, all with different targeting settings. Six of the adsets targeted the entire country, respectively. The daily budget for those six adsets was 168€. Beyond that, in Ghana, Kenya, and South Africa twelve additional adsets were used per country, each targeting parts of the country's population. Those adsets were constructed based on age (18-35 years, 36-65+), sex (male, female), and region (each country was divided into three regions), whereby each adset was budgeted with 14€ per day, also amounting to a daily budget of 168€. The ads themselves included one of five images, as well as an add text ("Are you 18+ and live in [Country Name]? Participate in our survey for a chance to win 5GB of mobile data!"), and a survey link. The ads were distributed over seven weeks (from Feb 7 to Mar 22, 2023). We implemented the targeted adsets only in three countries, as targeted adsets can increase the cost of a survey [1], and we are aware of only one prior study on whether targeting actually helps reducing survey bias [1]. Therefore, in a complementary manuscript within this project [2], the recruitment using targeted ads was compared against the recruitment where only one ad per country was used. This comparison showed that targeted adsets reduced only slightly the bias for univariate estimates, while bias was of relationships was similar across targeting strategies.

We used the same five images (see Figure S1) in all adsets, whereby Metas algorithm automatically decided how to distribute the images to maximize the number of clicks for the respective ads, which could not be influenced manually. Although we implemented a link parameter to know the image every participant saw before clicking on the link, this parameter was not given for all participants, as some removed the parameter. However, for those we know, 50.5% came through Image 1, 11.3% through Image 2, 8.3% through Image 3, 7.6% through Image 4, and 22.2% through Image 5 (see Table S1). This was somewhat different across countries. For example, in Tanzania Image 5 resulted in the most completes, whereas the image was only on third place in Nigeria. Regarding the composition of the sample by image, an investigation would not lead to meaningful conclusions given available data. To compare the effects of individual images on the sample composition, one would have to distribute the images in separate adsets as the Meta algorithm automatically optimizes within but not across adsets. Such an approach using separate adsets was implemented previously [3].

Compensation

As an incentive to participate in the AHS (including the vignette experiment), two respondents per country were randomly selected to receive 5GB of mobile data.

S2: Additional Tables and Figures

Table S1. Proportion of Respondents by Country through each Image

Country	Image 1	Image 2	Image 3	Image 4	Image 5
Ghana	49.16%	10.06%	7.26%	14.15%	19.37%
Kenya	55.61%	10.63%	8.43%	5.61%	19.73%
Nigeria	66.22%	14.82%	1.52%	4.66%	12.79%
South Africa	41.71%	13.59%	11.75%	5.07%	27.88%
Tanzania	24.61%	3.13%	2.91%	8.50%	60.85%
Uganda	32.62%	13.18%	14.19%	14.46%	25.55%
All	50.53%	11.35%	8.29%	7.60%	22.23%

Note: Proportions are based on all Survey Participants who completed the survey and did provide the link-parameter needed to know the image they saw in the Facebook ad (n=8.866).

Table S2. Variable Construction from AHS Data

Variables	Description
Attention Checks	
Passed Both Attention Checks	Respondent passed two attention checks; binary (1=yes, 0=no)
Passed One Attention Check	Respondent passed at least one attention check; binary (1=yes, 0=no)
Age	Age was measured and kept metric
Cognitive Skills	Number of correctly answered cognitive mathematical questions (0=no question answered correctly,1=one question answered correctly, 2=two questions answered correctly, 3=all three questions answered correctly)
Concerns & Risk	
Blood Test Concerns	The extent of concern about ethics and save blood-test-data handling; ordinal (1=not at all concerned, 2=just a little concerned, 3=somewhat concerned, 4=mostly concerned, 5=very concerned)
Risk Taking	Respondent is generally willing to take risk; ordinal (1=completely unwilling to take risks, 2=unwilling to take risks, 3=somewhat unwilling to take risks, 4=neutral, 5=somewhat willing to take risks, 6=willing to take risks, 7=very willing to take risks)
Country	
Ghana	Respondent lives in Ghana; binary (1=yes, 0=no)
Kenya	Respondent lives in Kenya; binary (1=yes, 0=no)
Nigeria	Respondent lives in Nigeria; binary (1=yes, 0=no)
South Africa	Respondent lives in South Africa; binary (1=yes, 0=no)
Tanzania	Respondent lives in Tanzania; binary (1=yes, 0=no)
Uganda	Respondent lives in Uganda; binary (1=yes, 0=no)
Education	
Secondary Education	Highest degree is secondary education; binary (1=yes, 0=no)
Tertiary Education	Highest degree is tertiary education; binary (1=yes, 0=no)
Primary Education	Highest degree is primary education; binary (1=yes, 0=no)
Female	Respondent is female; binary (1=yes, 0=no)
Has Children	Respondent has one or more children; binary (1=yes, 0=no)

Note: Construction of Variables used in the Regression Models

Table S2. Continued

Variables	Description
Health	
Diabetes Diagnosed	Respondent had diabetes diagnosed; binary (1=yes, 0=no)
General Health	Self-evaluation of general health; ordinal (1=very poor, 2=poor, 3=somewhat poor, 4=neither good nor poor, 5=somewhat good, 6=good, 7=excellent
HIV Positive	Respondent is HIV positive; binary (1=yes, 0=no)
Marital Status	
Divorced	Respondent is divorced, widowed or separated; binary (1=yes, 0=no)
Living together	Respondent lives together with a partner; binary (1=yes, 0=no)
Married	Respondent is married; binary (1=yes, 0=no)
Single	Respondent is single; binary (1=yes, 0=no)
Trust	
Social Trust	Respondent sees himself as generally trusting; ordinal (1=strongly disagree, 2=disagree, 3=somewhat disagree, 4=neither disagree nor agree, 5=somewhat agree, 6=agree, 7=strongly agree)
Trust in Science	Science and research are trustworthy; ordinal (1=not at all trustworthy; 2=just a little trustworthy; 3=somewhat trustworthy; 4=a lot trustworthy
Trust Ministry	Amount of trust respondent has towards the ministry in his country; ordinal (1=no trust at all, 2=hardly trust, 3=just a little trust, 4=somewhat trust, 5=mostly trust, 6=trust a lot, 7=complete trust)
Trust NGO	Amount of trust respondent has towards humanitarian organization unrelated to the government; ordinal (1=no trust at all, 2=hardly trust, 3=just a little trust, 4=somewhat trust, 5=mostly trust, 6=trust a lot, 7=complete trust)
Trust Statistical Office	Amount of trust respondent has towards the national statistical office in his country; ordinal (1=no trust at all, 2=hardly trust, 3=just a little trust, 4=somewhat trust, 5=mostly trust, 6=trust a lot, 7=complete trust)
Trust University	Amount of trust respondent has towards local universities; ordinal (1=no trust at all, 2=hardly trust, 3=just a little trust, 4=somewhat trust, 5=mostly trust, 6=trust a lot, 7=complete trust)
Survey Burden	
Survey Enjoyment	Respondent enjoys participating in online questionnaires; ordinal (1=strongly disagree, 2=disagree, 3=somewhat disagree, 4=neither disagree nor agree, 5=somewhat agree, 6=agree, 7=strongly agree)
Survey Value	Respondent judges surveys as important for society; ordinal; (1=strongly disagree, 2=disagree, 3=somewhat disagree, 4=neither disagree nor agree, 5=somewhat agree, 6=agree, 7=strongly agree)
Wealth	Self-evaluation of wealth compared to others in the country; ordinal (1=very poor, 2=poor, 3=average, 4=rich, 5=very rich)

Note: Construction of Variables used in the Regression Models

Table S3. Descriptive Table for All Additional Variables

Variables	Mean	Median	Min	Max	SD
Attention Checks					
Passed Both Attention Checks	0.755	1	0	1	0.430
Passed One Attention Check	0.956	1	0	1	0.206
Concerns & Risk					
Blood Test Concerns	4.039	4	1	5	1.170
Risk Taking	5.564	6	1	7	1.266
Trust					
Social Trust	5.506	6	1	7	1.402
Trust in Science	3.405	4	1	4	0.723
Trust Ministry	4.992	5	1	7	1.516
Trust NGO	5.053	5	1	7	1.381
Trust Statistical Office	4.658	5	1	7	1.485
Trust University	4.808	5	1	7	1.357
Health					
Diabetes Diagnosed	0.100	0	0	1	0.301
General Health	5.708	6	1	7	1.079
HIV Positive	0.030	0	0	1	0.170
Survey Burden					
Survey Enjoyment	5.827	6	1	7	1.352
Survey Value	6.178	6	1	7	1.177
Wealth	2.915	3	1	5	0.600
Cognitive Skills	2.194	2	0	3	0.836
Education					
Primary Education	0.011	0	0	1	0.102
Secondary Education	0.249	0	0	1	0.432
Tertiary Education	0.741	1	0	1	0.438
Age	29.420	27	18	97	8.935
Female	0.346	0	0	1	0.476
Has Children	0.537	1	0	1	0.499
Marital Status					
Divorced	0.028	0	0	1	0.164
Living together	0.079	0	0	1	0.270
Married	0.343	0	0	1	0.475
Single	0.550	1	0	1	0.498
Country					
Ghana	0.054	0	0	1	0.226
Kenya	0.574	1	0	1	0.495
Nigeria	0.129	0	0	1	0.335
South Africa	0.043	0	0	1	0.202
Tanzania	0.048	0	0	1	0.215
Uganda	0.152	0	0	1	0.359

Note: Descriptives for all variables not part of the vignette.

Table S4. Determinants of Participation in Survey or Blood Test (OLS): Main Effects and Hypotheses (Related to Figure 1)

	(1)		(2)		(3)	
	b	SE	b	SE	b	SE
Blood Test[a]	-0.021	(0.015)	-0.021	(0.015)	0.035	(0.056)
HIV[b]	0.010	(0.022)	0.003	(0.022)	0.002	(0.023)
NGO[c]	0.042	(0.032)	0.046	(0.031)	-0.070	(0.135)
Stat. Office[c]	0.041	(0.031)	0.050	(0.031)	-0.074	(0.125)
Ministry[c]	0.063*	(0.031)	0.073*	(0.031)	0.055	(0.126)
US $2 Incentive[d]	0.188***	(0.032)	0.184***	(0.031)	0.183***	(0.031)
US $2 Voucher[d]	0.200***	(0.032)	0.199***	(0.031)	0.198***	(0.031)
Chance[d]	0.180***	(0.032)	0.176***	(0.031)	0.177***	(0.031)
Trust NGO			0.086***	(0.012)	0.068***	(0.013)
Trust Stat. Office			0.022	(0.014)	0.001	(0.015)
Trust Ministry			0.048***	(0.013)	0.033*	(0.014)
Trust University			0.015	(0.013)	0.0003	(0.014)
HIV Positive			0.296***	(0.070)	0.278**	(0.100)
Diabetes Diagnosed			0.135**	(0.042)	0.130*	(0.053)
Trust Science			0.128***	(0.021)	0.127***	(0.021)
Trust Social			0.037***	(0.010)	0.038***	(0.010)
Blood Test Concerns			0.034**	(0.012)	0.041**	(0.013)
Blood Test[a]: Blood Test Concerns					-0.013	(0.013)
NGO[c]:Trust Ngo					0.076***	(0.021)
Stat. Office[c]:Trust Stat. Office					0.084***	(0.020)
Ministry[c]:Trust Ministry					0.057**	(0.019)
University:Trust University					0.055*	(0.022)
HIV[b]:HIV Positive					0.037	(0.118)
Diabetes[e]:Diabetes Diagnosed					0.013	(0.070)
Constant	5.421***	(0.066)	3.810***	(0.115)	3.844***	(0.141)
N	21176		21176		21176	
Adj. R-Squared	0.0059		0.0356		0.0368	

Note: Results are obtained from OLS estimations. All regression specifications included country fixed effects. Left-out reference categories are as follows: [a] = Diabetes, [b] = Survey, [c] = University, [d] = No Incentive, [e] = HIV. Standard errors are clustered at the respondent level. †p<0.10; *p<0.05; **p<0.01; ***p<0.001.

Table S5. Determinants of Participation in Survey or Blood Test (Ordered Logit Model): Robustness Check

	(1)		(2)		(3)	
	b	SE	b	SE	b	SE
Blood Test[a]	-0.027	(0.025)	-0.028	(0.026)	0.048	(0.091)
HIV[b]	0.027	(0.025)	0.013	(0.026)	0.018	(0.027)
NGO[c]	0.031	(0.036)	0.042	(0.036)	-0.106	(0.147)
Stat. Office[c]	0.011	(0.036)	0.030	(0.036)	-0.162	(0.136)
Ministry[c]	0.067†	(0.036)	0.095**	(0.036)	-0.015	(0.138)
US $2 Incentive[d]	0.228***	(0.036)	0.231***	(0.036)	0.230***	(0.036)
US $2 Voucher[d]	0.253***	(0.036)	0.259***	(0.036)	0.259***	(0.036)
Chance[d]	0.239***	(0.036)	0.239***	(0.036)	0.241***	(0.036)
Trust NGO			0.162***	(0.011)	0.140***	(0.013)
Trust Stat. Office			0.041**	(0.013)	0.012	(0.014)
Trust Ministry			0.079***	(0.012)	0.055***	(0.013)
Trust University			0.062***	(0.012)	0.043**	(0.014)
HIV Positive			0.420***	(0.080)	0.473***	(0.112)
Diabetes Diagnosed			0.201***	(0.044)	0.188**	(0.062)
Trust Science			0.169***	(0.019)	0.168***	(0.019)
Trust Social			0.066***	(0.009)	0.067***	(0.009)
Blood Test Concerns			0.089***	(0.011)	0.099***	(0.016)
Blood Test[a]: Blood Test Concerns					-0.019	(0.022)
NGO[c]:Trust Ngo					0.098***	(0.024)
Stat. Office[c]:Trust Stat. Office					0.116***	(0.022)
Ministry[c]:Trust Ministry					0.091***	(0.021)
University:Trust University					0.071**	(0.024)
HIV[b]:HIV Positive					-0.111	(0.159)
Diabetes[e]:Diabetes Diagnosed					0.027	(0.087)
N	21176		21176		21176	
Log Likelihood	-30,359.850		-29,605.220		-29,583.630	

Note: Results are obtained from ordered logit regressions and shows log odds. All regression specifications included country fixed effects. Left-out reference categories are as follows: [a] = Diabetes, [b] = Survey, [c] = University, [d] = No Incentive, [e] = HIV. Standard errors are clustered at the respondent level. †p<0.10; *p<0.05; **p<0.01; ***p<0.001.

Table S6. Determinants of Participation in Survey or Blood test (OLS): Robustness Check Including Socio-Demographic Controls

	(1)		(2)		(3)	
	b	SE	b	SE	b	SE
Blood Test[a]	-0.021	(0.015)	-0.021	(0.015)	0.035	(0.056)
HIV[b]	0.013	(0.022)	0.005	(0.021)	0.005	(0.023)
NGO[c]	0.037	(0.031)	0.041	(0.031)	-0.081	(0.135)
Stat. Office[c]	0.038	(0.031)	0.046	(0.031)	-0.081	(0.125)
Ministry[c]	0.060†	(0.031)	0.071*	(0.031)	0.048	(0.126)
US $2 Incentive[d]	0.183***	(0.032)	0.179***	(0.031)	0.178***	(0.031)
US $2 Voucher[d]	0.202***	(0.031)	0.202***	(0.031)	0.201***	(0.031)
Chance[d]	0.180***	(0.032)	0.177***	(0.031)	0.177***	(0.031)
Trust NGO			0.086***	(0.012)	0.069***	(0.013)
Trust Stat. Office			0.024†	(0.014)	0.004	(0.015)
Trust Ministry			0.050***	(0.013)	0.035*	(0.014)
Trust University			0.018	(0.013)	0.004	(0.014)
HIV Positive			0.261***	(0.070)	0.235*	(0.100)
Diabetes Diagnosed			0.060	(0.042)	0.052	(0.053)
Trust Science			0.123***	(0.021)	0.123***	(0.021)
Trust Social			0.037***	(0.010)	0.038***	(0.010)
Blood Test Concerns			0.034**	(0.012)	0.040**	(0.013)
Blood Test[a]: Blood Test Concerns					-0.013	(0.013)
NGO[c]:Trust Ngo					0.075***	(0.021)
Stat. Office[c]:Trust Stat. Office					0.082***	(0.020)
Ministry[c]:Trust Ministry					0.056**	(0.019)
University:Trust University					0.053*	(0.022)
HIV[b]:HIV Positive					0.051	(0.117)
Diabetes[e]:Diabetes Diagnosed					0.018	(0.070)
Age	0.001	(0.002)	0.004*	(0.002)	0.004*	(0.002)
Female	0.032	(0.029)	0.055†	(0.028)	0.056†	(0.028)
Secondary Education	0.173	(0.120)	0.061	(0.119)	0.058	(0.120)
Tertiary Education	0.202†	(0.117)	0.078	(0.117)	0.074	(0.118)
Wealth	-0.009	(0.023)	-0.038†	(0.023)	-0.038†	(0.023)
Living Together	-0.066	(0.054)	-0.018	(0.054)	-0.016	(0.054)
Married	-0.002	(0.041)	-0.031	(0.041)	-0.031	(0.041)
Divorced	-0.027	(0.089)	-0.028	(0.086)	-0.025	(0.086)
Child	0.258***	(0.041)	0.235***	(0.041)	0.233***	(0.041)
Constant	5.096***	(0.161)	3.597***	(0.181)	3.635***	(0.199)
N	21176		21176		21176	
Adj. R-Squared	0.0124		0.0422		0.0433	

Note: Results are obtained from OLS estimations. All regression specifications included country fixed effects and compared to the baseline specification additional controls. Left-out reference categories are as follows: [a] = Diabetes, [b] = Survey, [c] = University, [d] = No Incentive, [e] = HIV. Standard errors are clustered at the respondent level. †p<0.10; *p<0.05; **p<0.01; ***p<0.001.

Table S7. Determinants of Participation in Survey or Blood Test (OLS): Robustness Check Including Covariate Selected Based on Double Machine Learning Including Lasso Regression Partialing-Out

	(1)		(2)		(3)	
	b	SE	b	SE	b	SE
Blood Test[a]	-0.02	(0.015)	-0.02	(0.015)	0.028	(0.056)
HIV[b]	0.009	(0.021)	0.005	(0.021)	0.007	(0.023)
NGO[c]	0.044	(0.031)	0.045	(0.031)	-0.174	(0.113)
Stat. Office[c]	0.04	(0.03)	0.045	(0.03)	-0.172	(0.105)
Ministry[c]	0.064*	(0.031)	0.071*	(0.031)	-0.044	(0.106)
US $2 Incentive[d]	0.187***	(0.031)	0.181***	(0.031)	0.183***	(0.031)
US $2 Voucher[d]	0.205***	(0.031)	0.202***	(0.031)	0.202***	(0.031)
Chance[d]	0.181***	(0.031)	0.177***	(0.031)	0.176***	(0.031)
Trust NGO			0.058***	(0.012)	0.046**	(0.013)
Trust Stat. Office			0.022	(0.014)	0.01	(0.015)
Trust Ministry			0.041**	(0.013)	0.029*	(0.014)
Trust University			0.005	(0.013)	-0.006	(0.014)
HIV Positive			0.298***	(0.068)	0.268**	(0.1)
Diabetes Diagnosed			0.109*	(0.042)	0.103†	(0.053)
Trust Science			0.088***	(0.021)	0.089***	(0.021)
Trust Social			0.017†	(0.01)	0.015	(0.01)
Blood Test Concerns			0.002	(0.012)	0.009	(0.014)
Blood Test[a]: Blood Test Concerns					-0.015	(0.013)
NGO[c]:Trust Ngo					0.063**	(0.021)
Stat. Office[c]:Trust Stat. Office					0.071***	(0.02)
Ministry[c]:Trust Ministry					0.051**	(0.019)
University:Trust University					0.049*	(0.022)
HIV[b]:HIV Positive					0.09	(0.109)
Diabetes[e]:Diabetes Diagnosed					0.028	(0.069)
N	21176		21176		21176	

Note: Results are obtained from OLS estimations. All regression specifications included country fixed effects and additional covariates selected by double machine learning procedures based on a Lasso-learner. Left-out reference categories are as follows: [a] = Diabetes, [b] = Survey, [c] = University, [d] = No Incentive, [e] = HIV. Standard errors are clustered at the respondent level. †p<0.10; *p<0.05; **p<0.01; ***p<0.001.

Table S8. Determinants of Participation in Survey or Blood Test (OLS): Robustness Check Using Multi-Level Random Effects Model

	(1)		(2)		(3)	
	b	SE	b	SE	b	SE
Blood Test[a]	-0.021	(0.015)	-0.021	(0.015)	0.034	(0.054)
HIV[b]	-0.026	(0.019)	-0.029	(0.019)	-0.030	(0.020)
NGO[c]	0.021	(0.026)	0.024	(0.026)	-0.098	(0.105)
Stat. Office[c]	0.026	(0.026)	0.031	(0.026)	-0.122	(0.097)
Ministry[c]	0.054*	(0.026)	0.060*	(0.026)	-0.001	(0.099)
US $2 Incentive[d]	0.190***	(0.027)	0.188***	(0.026)	0.187***	(0.026)
US $2 Voucher[d]	0.196***	(0.026)	0.196***	(0.026)	0.195***	(0.026)
Chance[d]	0.165***	(0.026)	0.164***	(0.026)	0.164***	(0.026)
Trust NGO			0.086***	(0.012)	0.070***	(0.012)
Trust Stat. Office			0.022†	(0.013)	0.002	(0.014)
Trust Ministry			0.048***	(0.012)	0.033*	(0.013)
Trust University			0.015	(0.012)	0.003	(0.013)
HIV Positive			0.295***	(0.079)	0.283**	(0.095)
Diabetes Diagnosed			0.135**	(0.044)	0.137*	(0.054)
Trust Science			0.128***	(0.019)	0.128***	(0.019)
Trust Social			0.037***	(0.010)	0.037***	(0.010)
Blood Test Concerns			0.034**	(0.012)	0.040**	(0.013)
Blood Test[a]: Blood Test Concerns					-0.013	(0.013)
NGO[c]:Trust Ngo					0.068***	(0.017)
Stat. Office[c]:Trust Stat. Office					0.080***	(0.016)
Ministry[c]:Trust Ministry					0.057***	(0.015)
University:Trust University					0.047**	(0.017)
HIV[b]:HIV Positive					0.023	(0.109)
Diabetes[e]:Diabetes Diagnosed					-0.002	(0.062)
Constant	5.456***	(0.063)	3.843***	(0.109)	3.896***	(0.126)
N	21176		21176		21176	
Marginal R^2/Conditional R^2	0.006/0.525		0.006/0.525		0.037/0.526	

Note: Results are obtained from OLS estimations. All regression specifications included country fixed effects. Left-out reference categories are as follows: [a] = Diabetes, [b] = Survey, [c] = University, [d] = No Incentive, [e] = HIV. Standard errors are estimated based on a multilevel random effects model. †p<0.10; *p<0.05; **p<0.01; ***p<0.001.

Table S9. Determinants of Participation in Survey or Blood Test (OLS): Robustness Check Using a Reduced Analytical Sample (Attention Check Exclusion)

	(1)		(2)		(3)	
	b	SE	b	SE	b	SE
Blood Test[a]	-0.022	(0.015)	-0.022	(0.015)	0.017	(0.057)
HIV[b]	0.014	(0.022)	0.006	(0.022)	0.005	(0.023)
NGO[c]	0.049	(0.032)	0.056†	(0.032)	-0.104	(0.139)
Stat. Office[c]	0.036	(0.032)	0.044	(0.031)	-0.145	(0.127)
Ministry[c]	0.052	(0.032)	0.062†	(0.032)	0.004	(0.129)
US $2 Incentive[d]	0.187***	(0.032)	0.184***	(0.032)	0.184***	(0.032)
US $2 Voucher[d]	0.195***	(0.032)	0.193***	(0.032)	0.193***	(0.032)
Chance[d]	0.178***	(0.032)	0.172***	(0.032)	0.173***	(0.032)
Trust NGO			0.089***	(0.013)	0.071***	(0.014)
Trust Stat. Office			0.017	(0.015)	-0.005	(0.016)
Trust Ministry			0.050***	(0.014)	0.035*	(0.014)
Trust University			0.017	(0.014)	0.004	(0.014)
HIV Positive			0.279***	(0.075)	0.267*	(0.108)
Diabetes Diagnosed			0.159***	(0.042)	0.159**	(0.054)
Trust Science			0.118***	(0.021)	0.117***	(0.021)
Trust Social			0.039***	(0.010)	0.039***	(0.010)
Blood Test Concerns			0.036**	(0.012)	0.040**	(0.014)
Blood Test[a]:Blood Test Concerns					-0.009	(0.014)
NGO[c]:Trust Ngo					0.078***	(0.022)
Stat. Office[c]:Trust Stat. Office					0.091***	(0.020)
Ministry[c]:Trust Ministry					0.059**	(0.020)
University:Trust University					0.049*	(0.022)
HIV[b]:HIV Positive					0.026	(0.126)
Diabetes[e]:Diabetes Diagnosed					0.004	(0.072)
Constant	5.683***	(0.034)	2.959***	(0.144)	3.895***	(0.145)
N	20236		20236		20236	
Marginal R^2/Conditional R^2	0.0056		0.0348		0.0361	

Note: Table is based on a reduced analytical sample in which respondents who did not pass any of the two attention checks were dropped from the sample. Results are obtained from OLS estimations. All regression specifications included country fixed effects. Left-out reference categories are as follows: [a] = Diabetes, [b] = Survey, [c] = University, [d] = No Incentive, [e] = HIV. Standard errors are clustered at the respondent level. †p<0.10; *p<0.05; **p<0.01; ***p<0.001.

Table S10. Determinants of Participation in Survey or Blood Test (OLS): Robustness Check Using a Reduced Analytical Sample (7-Point Participation Scale Only)

	(1)		(2)		(3)	
	b	SE	b	SE	b	SE
Blood Test[a]	-0.016	(0.016)	-0.016	(0.016)	0.010	(0.059)
HIV[b]	0.032	(0.023)	0.026	(0.023)	0.028	(0.025)
NGO[c]	0.026	(0.034)	0.030	(0.033)	0.041	(0.147)
Stat. Office[c]	0.052	(0.033)	0.060†	(0.032)	0.024	(0.135)
Ministry[c]	0.047	(0.034)	0.057†	(0.033)	0.170	(0.137)
US $2 Incentive[d]	0.126***	(0.033)	0.125***	(0.033)	0.124***	(0.033)
US $2 Voucher[d]	0.149***	(0.033)	0.148***	(0.033)	0.148***	(0.033)
Chance[d]	0.140***	(0.034)	0.132***	(0.033)	0.132***	(0.033)
Trust NGO			0.080***	(0.013)	0.063***	(0.014)
Trust Stat. Office			0.033*	(0.015)	0.012	(0.016)
Trust Ministry			0.045**	(0.014)	0.032*	(0.015)
Trust University			0.009	(0.014)	-0.011	(0.015)
HIV Positive			0.227**	(0.075)	0.217*	(0.108)
Diabetes Diagnosed			0.128**	(0.045)	0.114*	(0.057)
Trust in Science			0.126***	(0.022)	0.125***	(0.022)
Social Trust			0.040***	(0.011)	0.040***	(0.011)
Blood Test Concerns			0.030*	(0.012)	0.033*	(0.014)
Blood Test[a]: Blood Test Concerns					-0.006	(0.014)
NGO[c]:Trust Ngo					0.071**	(0.023)
Stat. Office[c]:Trust Stat. Office					0.088***	(0.021)
Ministry[c]:Trust Ministry					0.051*	(0.020)
University[c]:Trust University					0.077***	(0.023)
HIV[b]:HIV Positive					0.022	(0.128)
Diabetes[e]:Diabetes Diagnosed					0.029	(0.075)
Constant	5.449***	(0.068)	3.866***	(0.123)	3.829***	(0.152)
N	17520		17520		17520	
Adj. R-Squared	0.0061		0.036		0.0373	

Note: Tables are based on a reduced analytical sample in which respondents where the likelihood of participation was asked on a 7-point Likert-scale. Results are obtained from OLS estimations. All regression specifications included country fixed effects. Left-out reference categories are as follows: [a] = Diabetes, [b] = Survey, [c] = University, [d] = No Incentive, [e] = HIV. Standard errors are clustered at the respondent level.
†p<0.10; *p<0.05; **p<0.01; ***p<0.001

Table S11. Determinants of Participation in Survey or Blood Test (OLS): Interaction Effects Between Trust and Incentives (Related to Figure 2)

	NGO	NGO	Stat. Office	Stat. Office	Ministry	Ministry	University	University	Science	Science	Social	Social
	(1)	(2)	(3)	(4)	(5)	(6)	(7)	(8)	(9)	(10)	(11)	(12)
HIV[a]	-0.005	-0.005	0.054	0.055	0.018	0.018	-0.060	-0.060	0.006	0.006	0.012	0.013
	(0.043)	(0.043)	(0.043)	(0.043)	(0.043)	(0.043)	(0.045)	(0.045)	(0.022)	(0.022)	(0.022)	(0.022)
Blood Test[b]	-0.014	-0.014	-0.001	-0.001	0.020	0.020	-0.074†	-0.074†	-0.021	-0.020	-0.021	-0.020
	(0.043)	(0.043)	(0.043)	(0.043)	(0.043)	(0.043)	(0.044)	(0.044)	(0.022)	(0.022)	(0.022)	(0.022)
Incentive[c]	0.219***	0.390†	0.171***	0.414*	0.213***	-0.035	0.143**	0.573**	0.186***	0.319*	0.188***	0.353**
	(0.053)	(0.219)	(0.050)	(0.180)	(0.051)	(0.183)	(0.051)	(0.208)	(0.026)	(0.130)	(0.026)	(0.110)
Trust NGO	0.187***	0.212***										
	(0.017)	(0.037)										
Incentive[c]:Trust NGO		-0.034										
		(0.042)										
Trust Stat. Office			0.161***	0.201***								
			(0.016)	(0.032)								
Incentive[c]:Trust Stat. Office				-0.052								
				(0.037)								
Trust Ministry					0.148***	0.111***						
					(0.016)	(0.031)						
Incentive[c]:Trust Ministry						0.050						
						(0.036)						
Trust University							0.135***	0.202***				
							(0.018)	(0.036)				
Incentive[c]:Trust University								-0.089*				
								(0.041)				
Trust in Science									0.221***	0.250***		
									(0.016)	(0.033)		
Incentive[c]:Trust in Science										-0.039		
										(0.037)		
Social Trust											0.080***	0.103***
											(0.008)	(0.017)
Incentive[c]:Social Trust												-0.031
												(0.019)
Constant	4.207***	4.097***	4.767***	4.570***	4.289***	4.466***	4.292***	3.933***	4.509***	4.413***	4.736***	4.605***
	(0.290)	(0.328)	(0.246)	(0.285)	(0.304)	(0.337)	(0.299)	(0.339)	(0.144)	(0.174)	(0.142)	(0.165)
N	5250	5250	5285	5285	5307	5307	5334	5334	21176	21176	21176	21176
Adj. R^2	0.0313	0.0313	0.0287	0.0289	0.0267	0.027	0.0157	0.0165	0.0158	0.0158	0.0107	0.0108

Note: Results are obtained from OLS estimations. All regression specifications included country fixed effects. Standard errors are clustered at the respondent level.
[a] = Diabetes, [b] = University, [c] = No Incentive
†p<0.10; *p<0.05; **p<0.01; ***p<0.001

Table S12. Determinants of Participation in Survey or Blood Test (OLS): Additional Socio-Economic Controls (Related to Figure 3)

	Participation in Survey and Blood Test	
	b	SE
Risk Taking	0.077***	(0.012)
General Health	0.049***	(0.014)
Survey Enjoyment	0.128***	(0.013)
Survey Value	0.077***	(0.015)
Cognitive Skills	0.060***	(0.017)
Age	0.003†	(0.002)
Female	0.046	(0.029)
Secondary Educ	-0.056	(0.124)
Tertiary Educ	-0.066	(0.122)
Wealth	-0.037	(0.023)
Living Together	-0.053	(0.054)
Married	-0.018	(0.041)
Divorced	-0.012	(0.087)
Child	0.229***	(0.041)
Constant	3.345***	(0.190)
N	21176	
Adj. R-Squared	0.0454	

Note: Results are obtained from OLS estimations. All regression specifications included country fixed effects and additional binary variables relate to all vignettes (topic, sponsor, incentive) and task (survey vs. blood test). Standard errors are clustered at the respondent level. †p<0.10; *p<0.05; **p<0.01; ***p<0.001.

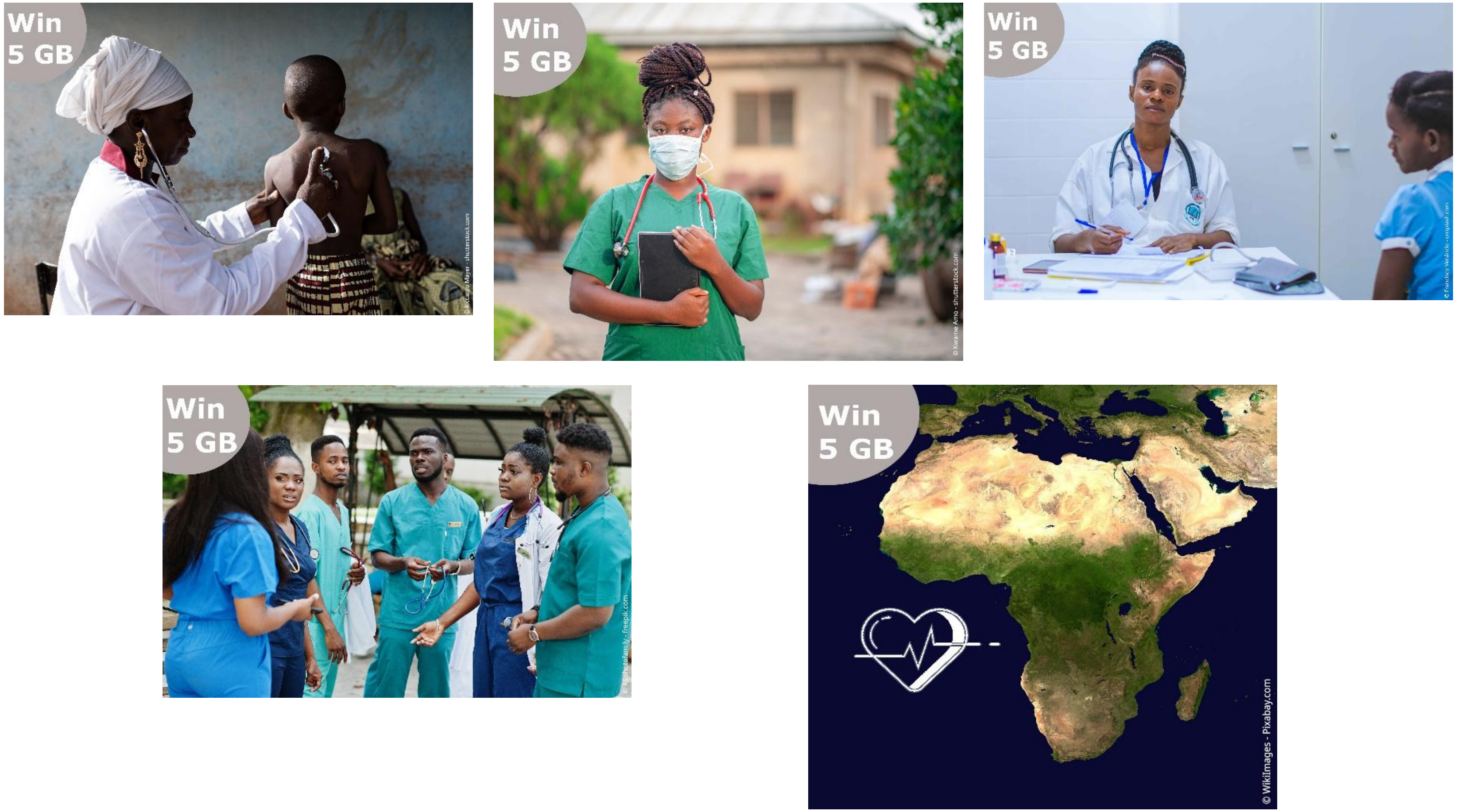

Figure S1. Images Used in the Facebook Advertisements

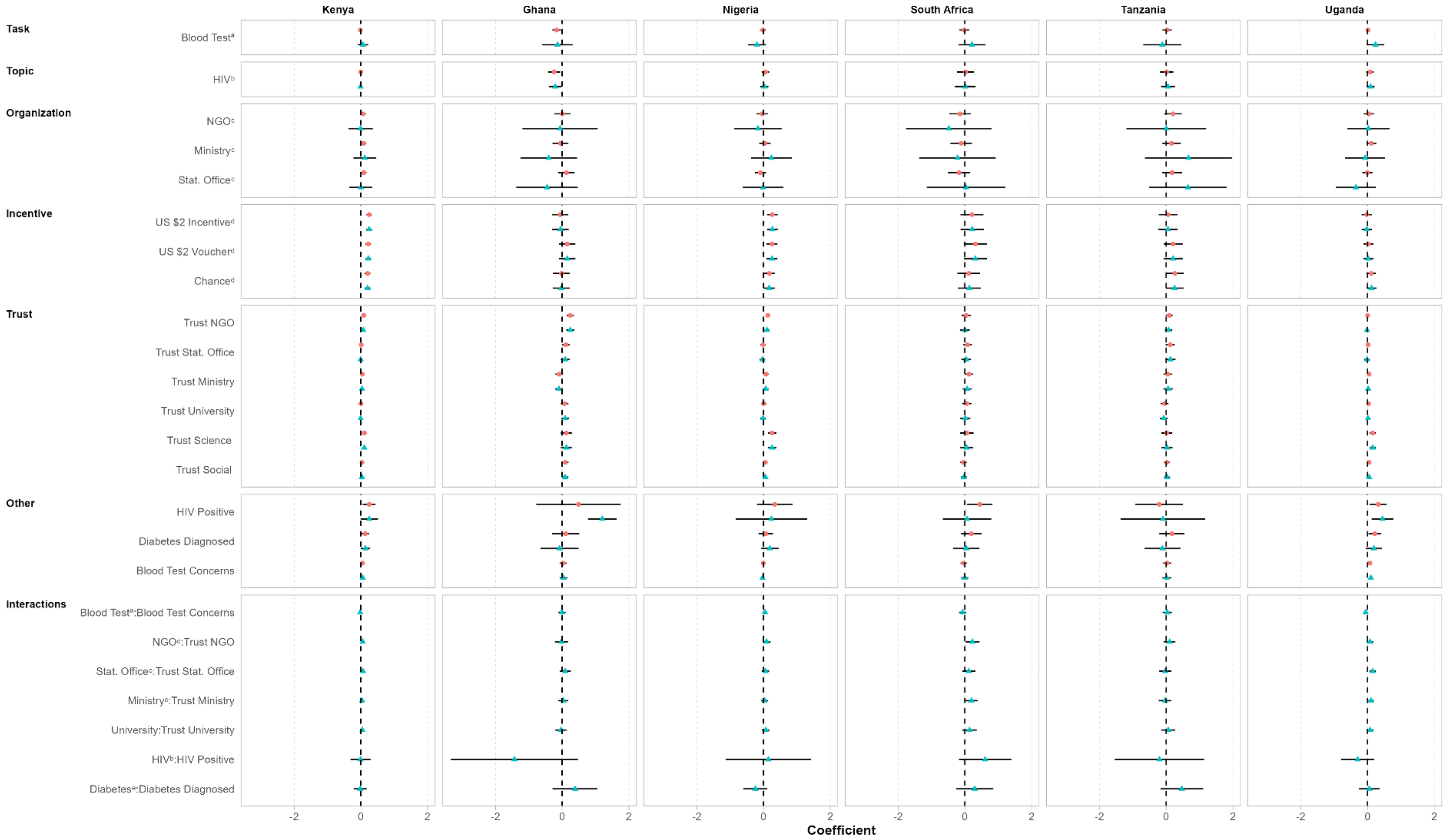

Figure S2. Determinants of Survey and Blood Test Participation (OLS): Main Effects and Hypotheses by Country

Note: Results are obtained from OLS estimations. The regressions a conducted separately by country. Model 1 (in red) includes displayed variables without any interaction effects, while Model 2 (in blue) includes interaction effects for trust and health status (HIV, diabetes) variables. Left-out reference categories are as follows: [a] = Survey, [b] = Diabetes, [c] = University, [d] = No Incentive, [e] = HIV. Standard errors are clustered at the respondent level. The displayed confidence intervals are at the 95% significance level. See Figure 1 for pooled.

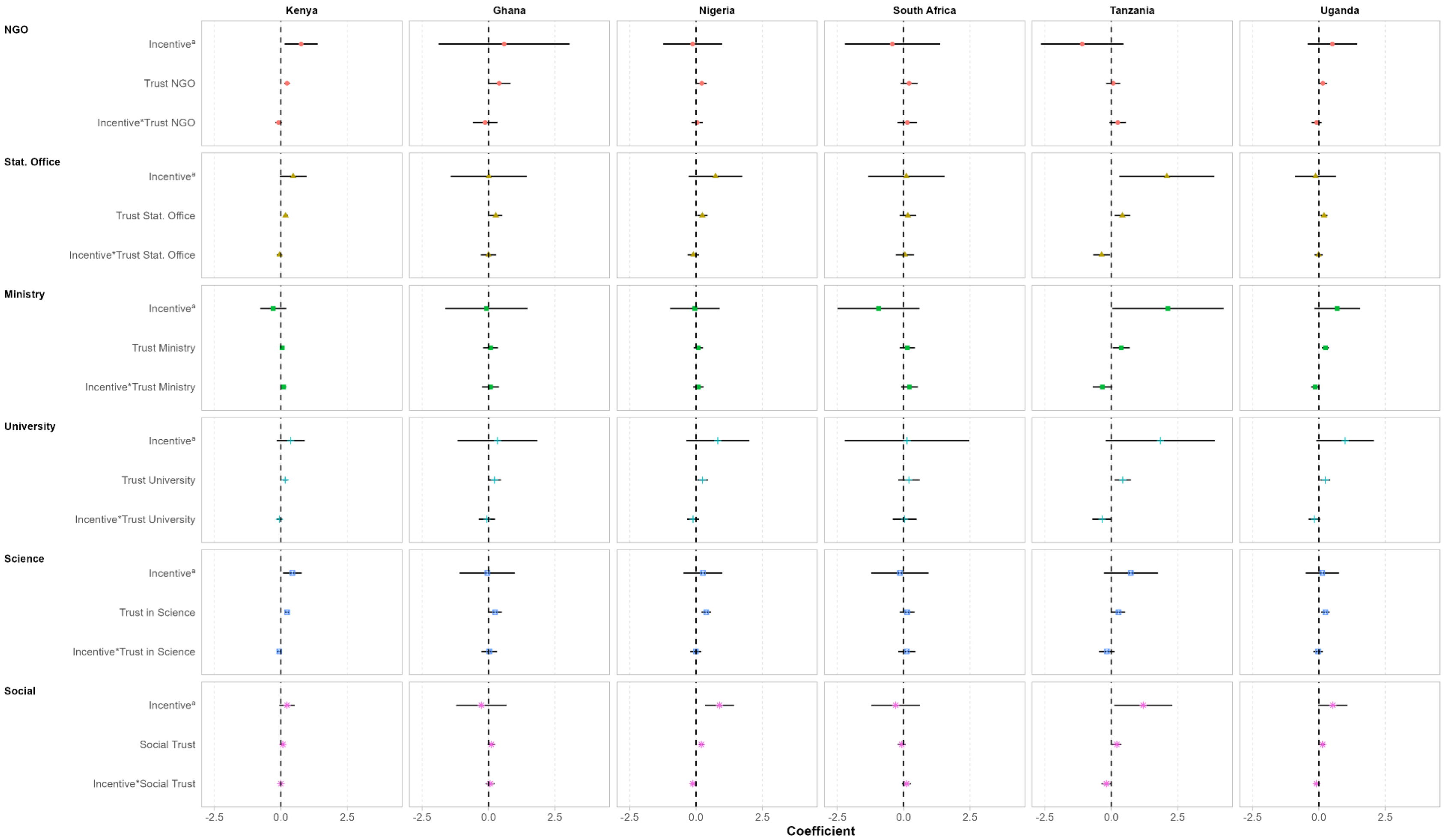

Figure S3. Determinants of Survey and Blood Test Participation (OLS): Interaction Between Trust in Institutions and Incentives by Country

Note: Results are obtained from OLS estimations. Estimations were run separately for each of the six trust measures and separately by country. The left-out category in each model is the 'no incentive' category. All regression specifications included the following covariates: binary variables on survey topic and task. In Standard errors are clustered at the respondent level. The displayed confidence intervals are at the 95% significance level. See Figure 2 for pooled results.

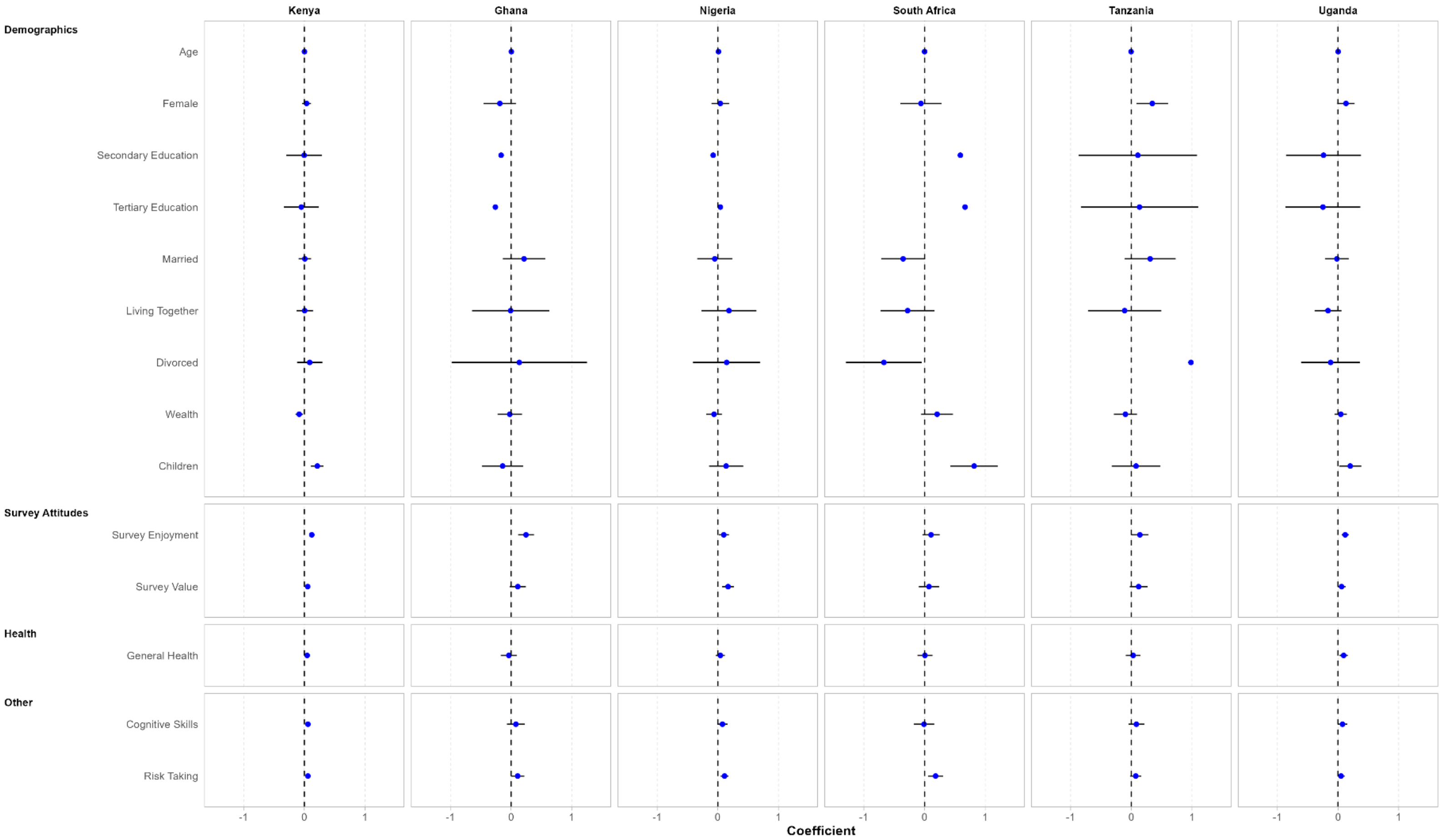

Figure S4. Determinants of Survey and Blood Test Participation (OLS): Role of Background Variables by Country

Note: Results are obtained from OLS estimations. All regressions are separately conducted by country. All regressions include the following covariates: binary variables on each vignette parameter (topic, sponsor, incentive) and task (survey vs. blood test). Standard errors are clustered at the respondent level. The displayed confidence intervals are at the 95% significance level. Confidence intervals for secondary and tertiary education were very wide for Ghana, Nigeria, and South Africa, as well as confidence intervals for divorce in Tanzania were very wide and are not displayed in the plot. See Figure 3 for pooled results.

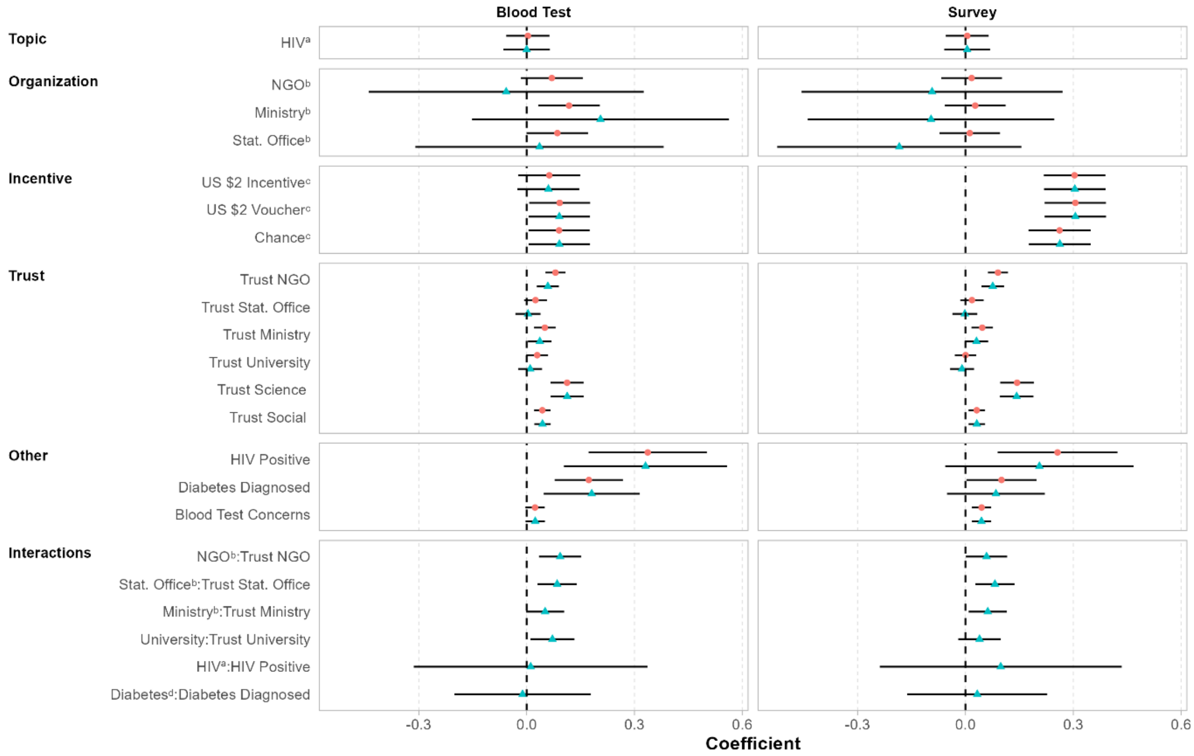

Figure S5. Determinants of Survey and Blood Test Participation (OLS): Main Effects and Hypotheses by Task

Note: Results are obtained from OLS estimations. The regressions are separately conducted by the Task. All regression specifications included country fixed effects. Model 1 (in red) includes displayed variables without any interaction effects, while Model 2 (in blue) includes interaction effects for trust and health status (HIV, diabetes) variables. Left-out reference categories are as follows: [a] = Diabetes, [b] = University, [c] = No Incentive, [d] = HIV. Standard errors are clustered at the respondent level. The displayed confidence intervals are at the 95% significance level, and Bonferroni adjusted to account for the testing of multiple hypotheses (8). See Figure 1 for pooled results.

References

1. Neundorf, A., & Öztürk, A. (2023). How to improve representativeness and cost-effectiveness in samples recruited through Meta: A comparison of advertisement tools. PLOS ONE, 18(2), e0281243. https://doi.org/10.1371/journal.pone.0281243
2. Rohr, B., Felderer, B., Silber, H., West, B.T., Pötzschke, S. & Priebe, J. (2025) Sampling for a Cross-National Survey in Six African Countries Using Social Media Advertisements: Comparison of Different Targeting Strategies, Types of Estimates, and Selectivity against Population Benchmarks, SocArXiv. https://doi.org/10.31235/osf.io/g9h47_v1
3. Neundorf, A., & Öztürk, A. (2022). Advertising Online Surveys on Social Media: How Your Advertisements Affect Your Samples, OSF Preprints. https://doi.org/10.31219/osf.io/84h3t
