# Supplementary material for: Incentives and Trust Are the Main Drivers of Recruiting Participants in 6 African Countries via Web-Based Environments: A Vignette Survey Experiment
Source: J Med Internet Res. 2025 Jun 25;27:e68472. doi: 10.2196/68472 (PMC12220199; doi:10.2196/68472)

Appendix C: CHERRIES CHECKLIST for the manuscript "Incentives and Trust Are Main Drivers of Recruiting Participants via Online Environments: A Vignette Experiment in Six African Countries"

Table 1. Checklist for Reporting Results of Internet E-Surveys (CHERRIES)

Item Category	*Checklist Item*	*Explanation*
**Design**		
	Describe survey design	The target population was any adult (18+ years) person living in Ghana, Kenya, Nigeria, South Africa, Tanzania, or Uganda. Respondents for the convenience sample were recruited via Facebook advertisements.
**IRB (Institutional Review Board) approval and informed consent process**		
	IRB approval	The project obtained IRB approval from the medical chamber of the state of Hamburg on November 30, 2022 (#2022-300276-WF) and from the GIGA institute on March 21, 2023 (#05/2023).
	Informed consent	Individuals who clicked on the survey link in the Facebook advertisements were directed to the survey landing page (see Figure C.1), where they were informed regarding the expected duration, investigators, purpose of the study, and received data protection information, with a link to a more detailed data protection letter (see Figure C.2). Only respondents who provided informed consent could go ahead by starting the survey (see Figure C.1).
	Data protection	The data are anonymized by design as no personal information was collected in the initial survey. Respondents who opted for a chance to win the 5GB mobile data incentive were directed to a second, unlinked survey, where they needed to provide an e-mail address and phone number. The respective e-mail addresses were used to contact the winners and ask if their phone number was correct and then after confirmation the numbers were used to give them the mobile data incentive. Afterward, all data from the second survey was deleted. All data from both surveys are or were stored on password-protected servers. The data collection fully complied with the General Data Protection Regulations of the EU (GDPR).

**Development and pre-testing**		
	Development and testing	The items were tested in a pretest in Sierra Leone. The technical functionality of the questionnaire was tested by the project team before data collection.
**Recruitment process and description of the sample having access to the questionnaire**		
	Open survey versus closed survey	The survey was open to anyone with the survey link.
	Contact mode	Respondents were invited online via advertisements on Facebook.
	Advertising the survey	The survey was advertised via Meta's advertisement platform on Facebook. See Figure C.3 for the five different advertisements used within the study.
**Survey administration**		
	Web/E-mail	The survey was conducted on the web survey platform Unipark as a self-administered web survey.
	Context	Unipark is a survey software tailored to academics, provided by the private company Tivian, where customers can design and host surveys.
	Mandatory/voluntary	The survey was voluntary. Participation could be stopped at any time.
	Incentives	All respondents were invited to take part in a lottery where they could win 5GB Mobile Data.
	Time/Date	The data collection started on February 7, 2023 and ended on March 22, 2023.
	Randomization of items or questionnaires	The survey included a fully randomized vignette experiment, which is analyzed for this manuscript.
	Adaptive questioning	Respondents could not receive the exact same vignette twice.
	Number of Items	The online survey comprised 119 items.
	Number of screens (pages)	The online survey displayed 44 screens.
	Completeness check	Sensitive questions contained a nonresponse/item refusal response category and selection of one response was enforced for all items.

	Review step	The survey did not include a back button or a review step.
**Response rates**		
	Unique site visitor	11,768,567
	View rate (Ratio of unique survey visitors/unique site visitors)	562,432 (site visitor) / 11,768,567 (ad shown) = 0.048
	Participation rate (Ratio of unique visitors who agreed to participate/unique first survey page visitors)	22,932 (started)/562,432 (site visitor) = 0.041
	Completion rate (Ratio of users who finished the survey/users who agreed to participate)	10,588 (completes) / 22,932 (started) = 0.462
**Preventing multiple entries from the same individual**		
	Cookies used	Unipark can use Cookies to identify unique users. Cookies were activated throughout the entire survey period. Cookies were set and read at the first survey screen (the screen following the landing page). Cookies were valid for 14 days.
	IP check	IP addresses & checks were not used or implemented to identify respondents or double entries.
	Log file analysis	We did not use log file analysis or other technics to identify multiple entries.
	Registration	Not applicable, since it was an open survey.
**Analysis**		
	Handling of incomplete questionnaires	In our analysis, we only used data from participants which gave answers to all items analyzed and removed participants with missing values, i.e., respondents who broke off before. Respondents that did not complete the whole questionnaire, but completed all questions analyzed were included. In total 10,588 individuals completed the survey at least up to the last item we included, while 12,344 broke off before and were excluded from our analysis.

	Questionnaires submitted with an atypical timestamp	We did not remove questionnaires based on time stamps. However, in a robustness check analysis reported in the manuscript (see Table A.9), we examined the impact of excluding respondents who did pass the included attention checks.
	Statistical correction	We did not perform statistical corrections, such as weighing or propensity score matching.

Figure C.1. Landing Page

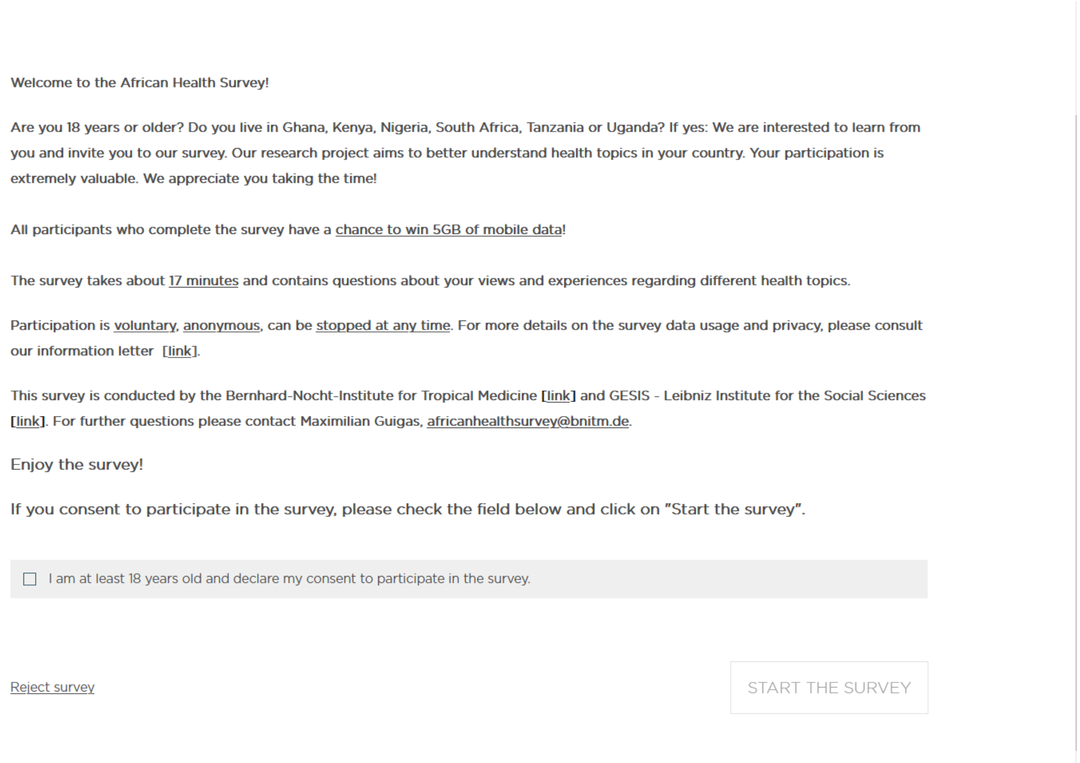

Welcome to the African Health Survey!

Are you 18 years or older? Do you live in Ghana, Kenya, Nigeria, South Africa, Tanzania or Uganda? If yes: We are interested to learn from you and invite you to our survey. Our research project aims to better understand health topics in your country. Your participation is extremely valuable. We appreciate you taking the time!

All participants who complete the survey have a chance to win 5GB of mobile data!

The survey takes about 17 minutes and contains questions about your views and experiences regarding different health topics.

Participation is voluntary, anonymous, can be stopped at any time. For more details on the survey data usage and privacy, please consult our information letter [link].

This survey is conducted by the Bernhard-Nocht-Institute for Tropical Medicine [link] and GESIS - Leibniz Institute for the Social Sciences [link]. For further questions please contact Maximilian Guigas, africanhealthsurvey@bnitm.de.

Enjoy the survey!

If you consent to participate in the survey, please check the field below and click on "Start the survey".

☐ I am at least 18 years old and declare my consent to participate in the survey.

Reject survey

START THE SURVEY

Figure C.2. Information letter on data protection and usage

BNITM
Bernhard Nocht Institute for Tropical Medicine

gesis
Leibniz Institute
for the Social Sciences

Information letter on data protection and usage in the online survey "African Health Survey"

The African Health Survey is run by the Bernhard-Nocht Institute for Tropical Medicine and the GESIS - Leibniz Institute for the Social Sciences. It serves research purposes only, namely to better understand public health and health-related behavior in African countries.

The survey contains questions on your sociodemographic background, social preferences, attitudes, emotions and behavior towards health-related topics.

Please be assured that we are not seeking any personal or identifiable information and that there is no analysis of individual data.

Responsibility

The survey is run by the Bernhard-Nocht Institute for Tropical Medicine and GESIS - Leibniz Institute for the Social Sciences. The principal investigators responsible for the content and handling of the data are Maximilian Guigas (BNITM) and Björn Rohr (GESIS).

Who can participate in the study

The target audience for this survey are all people who live in one of the following 6 countries: Ghana, Kenya, Nigeria, South Africa, Tanzania, Uganda. You must be at least 18 years old to participate in the study.

Participation in the lottery

Among all participants in each country who complete our survey, we will give away 5 GB of mobile data for your phone.
Please mind that not every participant will receive the 5 GB of mobile data, but 1 to 2 winners per country will be drawn randomly in a lottery.
To participate you have to leave your phone number and Email address at the end of the survey.
The winners are drawn randomly after the survey is over and will be notified via Email or text message to the submitted phone number or email address.
Each participant can enter the lottery only once to ensure equal chances for everyone.
Your phone number and Email address, which you have to submit to take part in the lottery, will be stored separately from the survey data to ensure anonymity.
Participation in the lottery is voluntary. If you do not want to leave your phone number you can still participate in the survey. However, participation in the lottery is not possible in this case.

What data are collected

We will collect self-reported answers to questions concerning your health-related attitudes, emotions and behavior. Your participation is completely voluntary, and you can stop the survey at any time if you are not comfortable with answering the questions.

Personal data

During this survey no personal information will be collected. However, if you want to participate in the lottery we ask you to provide your phone number and Email adress, which we will save until all prices are distributed. Participation in the lottery is voluntary!

Collection of technical data

Apart from the answers you give in the survey we will collect some technical data: date of participation, temporal duration of the survey, information on device and browser and the geographical location of your internet access based on your IP-address. The IP-address itself will not be collected.

Usage of data

Data will be collected using the survey software Unipark who complies with the General Data Protection Regulation (GDPR) of the EU. Further information on the software provider can be found on their website: https://www.unipark.com/.

The collected data will be analyzed anonymously as part of research projects. The data will be used for scientific purposes only. Data analysis includes the descriptive and graphical presentation and comparison as well as other statistical methods. Individual results will not be shared or reported in any way. We will only report grouped results. The final data set will be made publicly available to ensure transparent research methods.

Data protection

We employ several tools to ensure data security: First, the collected data will be stored on secure servers and they are password protected. Access to the data is limited to researchers that are working on the project. Data protection mechanisms from the survey platform provider Unipark can be found here: https://www.unipark.com/en/data-security/. We comply to the General Data Protection Regulation of the EU. The anonymous final data set will be made publicly available.

Your rights

- You may at any time request confirmation if and how involved researchers processes your personal data (Article 15 GDPR).
- You may have the right to receive a copy of your data in a structured, commonly used and machine-readable format so that you can further use or forward it to other organizations (Article 20 GDPR).
- If you are of the opinion that your personal data stored by the researchers is incorrect or incomplete, you may request us to rectify or complete such data (Article 16 GDPR).
- Under certain circumstances, you also have the right to object to the processing of your data (Article 21 GDPR) and you may request us to restrict our processing of your data (Article 18 GDPR) and erase your data (Article 17 GDPR).

There are some exceptions to these rights. For example, it is not possible to erase your data if researchers have a legal obligation to retain it or if the researchers are unable to identify your personal data.

If you are of the opinion that the processing of your personal data constitutes a breach of the GDPR or other applicable EU data protection law, you have the right to lodge a complaint with a data protection authority. The competent data protection authority is the Federal Commissioner for Data Protection and Freedom of Information.

- Postal address: Der Bundesbeauftragte für den Datenschutz und die Informationsfreiheit, Graurheindorfer Str. 153 - 53117 Bonn, Germany
- E-mail address: poststelle@bfdi.bund.de

In case you have questions, comments or concerns about this study or your participation you can contact us via Email to africanhealthsurvey@bnitm.de at any time.

Contact information

Principal investigator:

Maximilian Guigas
Bernhard-Nocht-Institute for Tropical Medicine | B118 | Bernhard-Nocht-Straße 74 | 20359 Hamburg
africanhealthsurvey@bnitm.de

Figure C.3. Example screenshots of the five Facebook advertisements used in Ghana

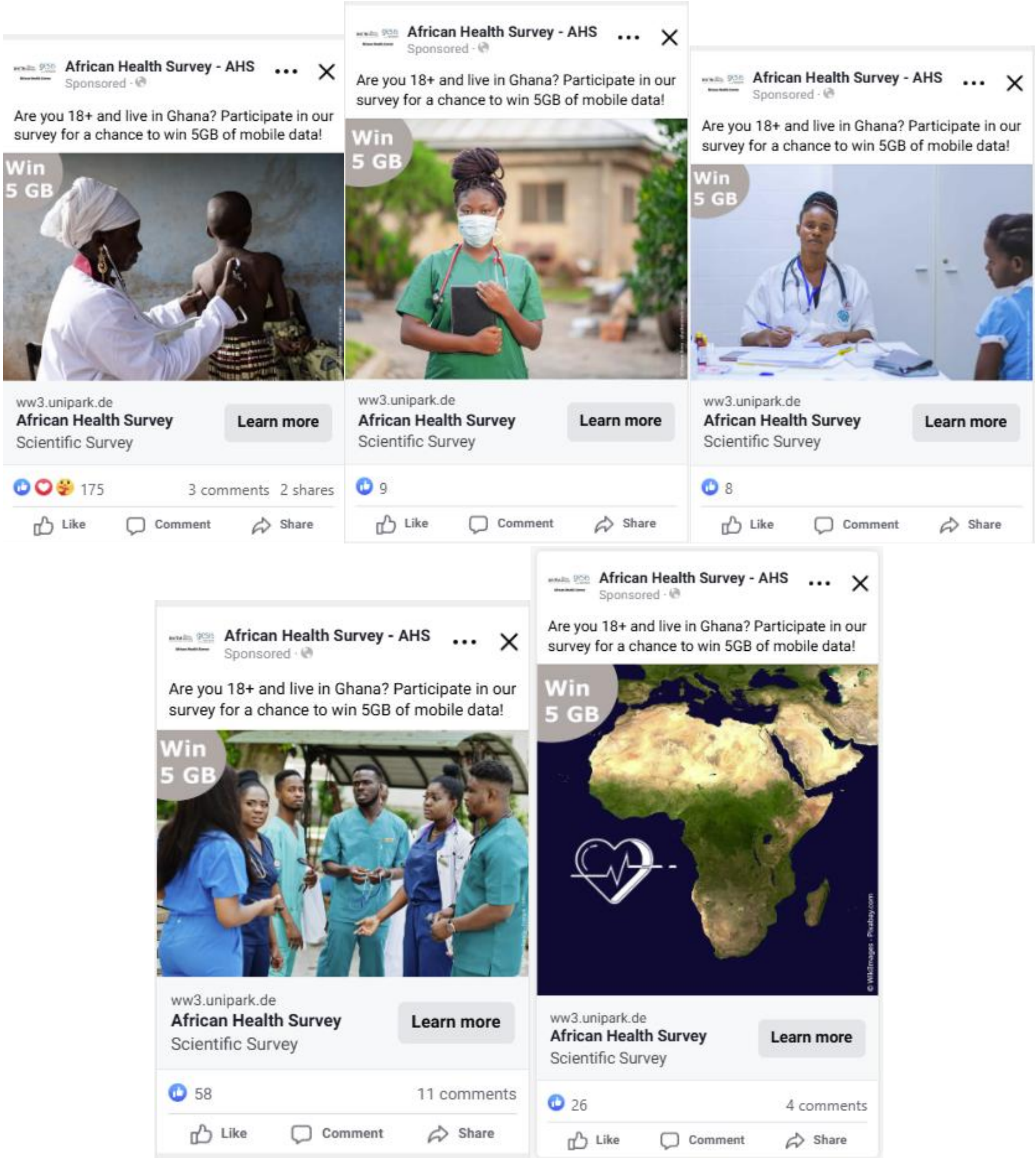

Supplement: Checklist 1 [file jmir-v27-e68472-s002.pdf]
